# The History of Tree and Shrub Taxa on Bol’shoy Lyakhovsky Island (New Siberian Archipelago) since the Last Interglacial Uncovered by Sedimentary Ancient DNA and Pollen Data

**DOI:** 10.3390/genes8100273

**Published:** 2017-10-13

**Authors:** Heike H. Zimmermann, Elena Raschke, Laura S. Epp, Kathleen R. Stoof-Leichsenring, Lutz Schirrmeister, Georg Schwamborn, Ulrike Herzschuh

**Affiliations:** 1 Alfred-Wegener-Institute Helmholtz Centre for Polar and Marine Research, Periglacial Research Unit, Telegrafenberg A43, 14473 Potsdam, Germany; elena.raschke@awi.de (E.R.); laura.epp@awi.de (L.S.E.); kathleen.stoof-leichsenring@awi.de (K.R.S.-L.); lutz.schirrmeister@awi.de (L.S.); georg.schwamborn@awi.de (G.S.); 2 Institute of Biochemistry and Biology, University of Potsdam, Karl-Liebknecht-Str. 24-25, 14476 Potsdam-Golm, Germany; 3 Institute of Earth and Environmental Sciences, University of Potsdam, Karl-Liebknecht-Str. 24-25, 14476 Potsdam-Golm, Germany

**Keywords:** sedaDNA, metabarcoding, trnL, single-nucleotide polymorphism (SNP), treeline, MIS 5 to 1, permafrost deposits, radiocarbon ages, palaeoenvironment, *Larix*

## Abstract

Ecosystem boundaries, such as the Arctic-Boreal treeline, are strongly coupled with climate and were spatially highly dynamic during past glacial-interglacial cycles. Only a few studies cover vegetation changes since the last interglacial, as most of the former landscapes are inundated and difficult to access. Using pollen analysis and sedimentary ancient DNA (*s*edaDNA) metabarcoding, we reveal vegetation changes on Bol’shoy Lyakhovsky Island since the last interglacial from permafrost sediments. Last interglacial samples depict high levels of floral diversity with the presence of trees (*Larix*, *Picea*, *Populus*) and shrubs (*Alnus*, *Betula*, *Ribes*, *Cornus,* Saliceae) on the currently treeless island. After the Last Glacial Maximum, *Larix* re-colonised the island but disappeared along with most shrub taxa. This was probably caused by Holocene sea-level rise, which led to increased oceanic conditions on the island. Additionally, we applied two newly developed larch-specific chloroplast markers to evaluate their potential for tracking past population dynamics from environmental samples. The novel markers were successfully re-sequenced and exhibited two variants of each marker in last interglacial samples. SedaDNA can track vegetation changes as well as genetic changes across geographic space through time and can improve our understanding of past processes that shape modern patterns.

## 1. Introduction

The Pleistocene epoch is characterised by glacial-interglacial cycles leading to pronounced changes of temperature and sea level [1,2,3], which in turn affect the prevailing ecosystems [4,5,6] and periglacial landscapes [7,8]. During glacial periods sea level was much lower than today, exposing the shallow arctic shelf, which extended the Eurasian continent northwards and connected it with the North American continent. Rapid climate warming at the onset of interglacial periods was accompanied by rising sea level [9,10,11] that submerged the shallow shelf systems. These climate-coupled dynamics affected species distributions, which responded by range contractions or expansions [12,13]. The spatial position of ecosystem boundaries accordingly changed through time. One such boundary is the Siberian treeline, also called the tundra-taiga ecotone, as it is a zone where boreal larch forest gradually changes into tundra [14]. The treeline is thought to have retreated southwards during glacial periods and to have shifted northwards during interglacial periods [5], but the northern limit of larch in Siberia during past interglacials is still unknown.

According to climate projections, the mean annual air temperature of the Siberian Arctic is expected to rise by about 4–7 °C by the year 2100 (B2 and A1 emissions scenarios [15]). This corresponds approximately to the reconstructed mean annual temperature of the last interglacial (Marine Isotope Stage (MIS) 5), which was about 5 °C higher than today [16]. The vegetation composition of MIS 5 could therefore putatively represent the future vegetation. Knowledge about the future vegetation is highly relevant in terms of potential vegetation-climate feedbacks that can cause polar amplification, for example through changes in albedo if forests expand into tundra [17,18,19,20,21,22] and if evergreen conifers displace deciduous larch forests [15,18]. Yet, forest vegetation of high northern latitudes may strongly lag climate change [23,24].

So far, few palaeoenvironmental studies cover the last interglacial period, especially in the Siberian high Arctic, because there are only a few locations known to have preserved sediments from that time. Bol’shoy Lyakhovsky Island (New Siberian Archipelago) is regarded as the most comprehensive permafrost archive in the Siberian Arctic, with deposits dating back to the middle Pleistocene [7]. It is therefore of high value for palaeoenvironmental reconstructions. Previous pollen- [4,7,25] and macrofossil-based [26] studies of outcrop sections determined that Bol’shoy Lyakhovsky was dominated by shrub tundra during MIS 5, as conifer-derived pollen was considered to be re-deposited and arboreal macrofossils were absent in the investigated samples.

Sedimentary ancient DNA (sedaDNA) has proven to be a valuable proxy for vegetation changes in periglacial settings [27,28,29,30] and is a promising tool to trace past local presences of organisms even though macrofossils or pollen may be absent [27,30,31]. *Sed*aDNA is predominantly local in origin and thought to be mostly derived from extracellular DNA from various plant tissues [29,32,33,34]. This is an advantage over pollen data, because different pollination strategies influence the amount of pollen production and pollen dispersal capacities [35,36]. Hence, comparisons of pollen data with vegetation surveys show that anemophilous taxa are usually over-represented and can be derived from extra-regional stands, while hydrophilous or entomophilous taxa are often under-represented [37,38,39,40]. A further advantage of sedaDNA over pollen is that sedaDNA allows for a higher proportion of taxa to be resolved on species level, especially in the Arctic [30,41]. However, it is recommended to use sedaDNA in combination with pollen or macrofossil records to ensure a more complete record of past vegetation, because differences due to taphonomy, taxonomic resolution and spatial scales can complement each other [30,33,42]. The contribution of pollen to the sedaDNA record is still challenging to quantify. The contribution is, however, regarded as low, since DNA from pollen has been difficult to amplify [43]. A significant advantage of sedaDNA over traditional pollen or macrofossil analyses is that it permits the analysis of past population dynamics within a species of interest through specific genetic markers or probes.

In this study we aim to track changes of the terrestrial plant community composition, in particular of tree and shrub taxa, at Bol’shoy Lyakhovsky from MIS 5 to MIS 1. We use plant-derived sedaDNA from permafrost sediments in a DNA metabarcoding approach and compare it with palynological analyses. DNA metabarcoding is a powerful method to assess the biodiversity of an environmental sample using high-throughput sequencing [42,44]. Here, we use the P6-loop of the chloroplast *trn*L (UAA) intron [45], which is a universal, plant-specific and short barcode marker, to enrich the samples for plant derived DNA prior to sequencing. We apply two newly developed larch-specific chloroplast markers to assess their potential to analyse past population dynamics in ancient sediments and to validate the presence of *Larix* sequences in the record.

## 2. Materials and Methods

### 2.1. Geographic Setting

**Figure 1:**
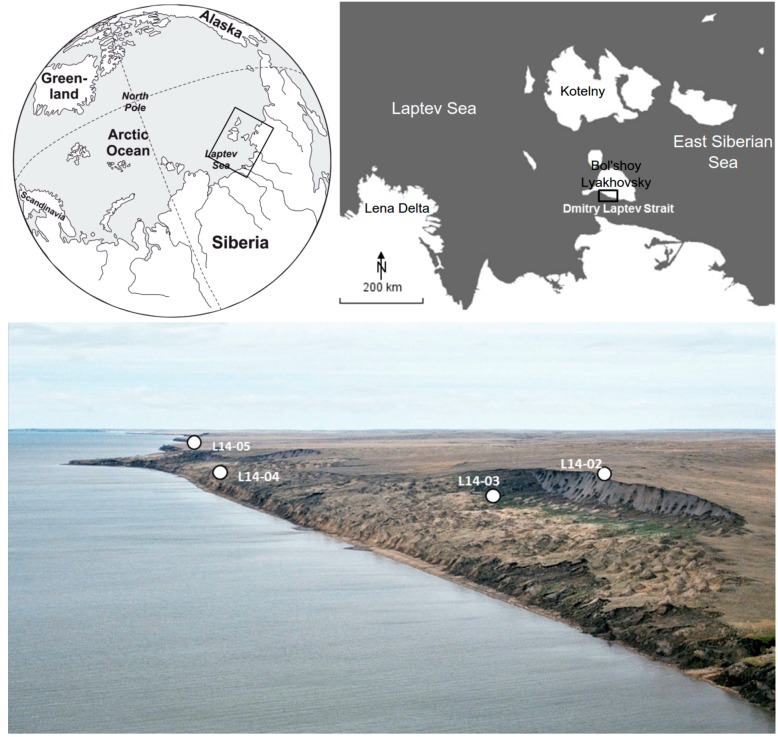
Map showing the location of Bol’shoy Lyakhovsky Island in the upper panel and an overview of the core drilling sites in an aerial photograph of the southern coast of the island (modified after [48]).

Bol’shoy Lyakhovsky Island is the southernmost island of the New Siberian Archipelago (Figure 1). It is bordered by the Dmitry Laptev Strait in the south, which connects the Laptev Sea in the west with the East Siberian Sea in the east. The island is underlain by continuous permafrost with an estimated thickness of 400–700 m [46], reflecting past and modern cold climatic conditions. During the last glacial the island was part of the Beringian landscape and not covered by permanent snow or an ice shield [47]. The seasonal thaw-depth (active layer) is shallow, reaching 0.6 m according to borehole measurements at the coring site L14-02 [48]. Mean annual temperature is −15 °C at the nearby station Cape Shalaurova [49]. The climate is characterised by short, cold summers with a mean July temperature of approximately 2.8 °C. The long, harsh winters last for eight months and are characterised by low light availability and low temperatures with a mean January temperature of −32.2 °C. Despite the maritime setting, annual precipitation is about 253 mm [49], yet overall conditions are humid due to low evaporation rates and because the permafrost table restricts the drainage of the wet active-layer during summer [26]. According to the Circumpolar Arctic Vegetation Map [50] the vegetation of Bol’shoy Lyakhovsky can be assigned to the rush/grass, forb, cryptogam tundra unit, the sedge/grass, moss wetland unit and the cryptogam, herb, barren unit.

### 2.2. Core Material

**Figure 2:**
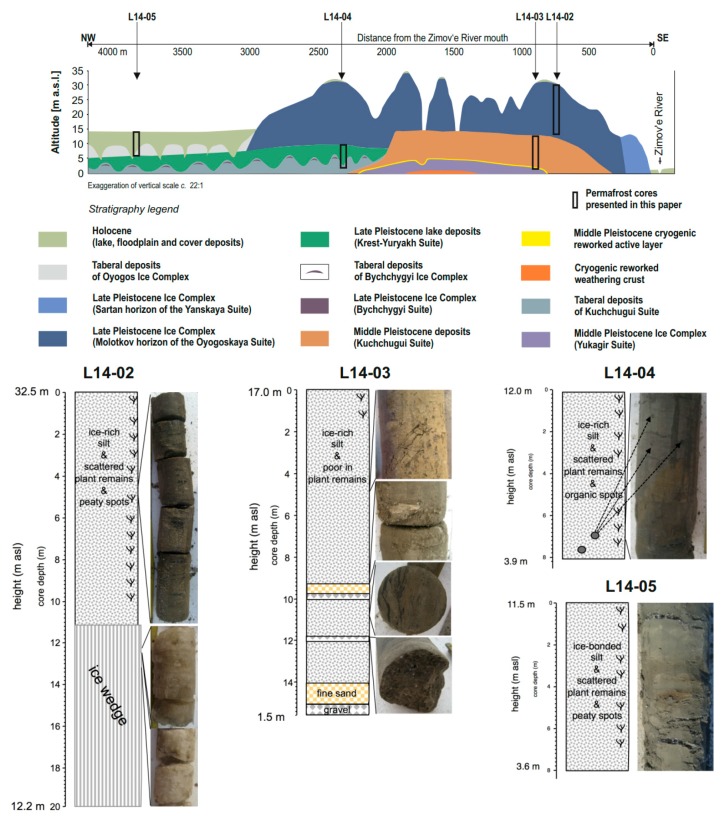
Quaternary stratigraphy of the southern coast of Bol’shoy Lyakhovsky Island with core positions indicated by black boxes [48] (modified after [4,8,25]). The lower panel shows the schematic core recoveries with photographic examples [48].

Permafrost coring took place at the southern coast of Bol’shoy Lyakhovsky, west of Zimov’e River in April 2014 [48]. Permafrost records often do not reflect the full sequence of Quaternary periods due to complex periglacial processes [51]. Hence, sediments of different ages and accumulation types are located at different positions and elevations [51] and are exposed on steep bluffs of thermokarst depressions, thermo-erosional valleys and Yedoma hills [7]. Based on stratigraphic knowledge from previous studies [4,7,8,25,52], coring was undertaken at four localities to obtain a record spanning from the last interglacial to the Holocene (MIS 5–MIS 1). The cores were transported and stored continuously frozen. The placement of the cores within the local stratigraphy is shown in Figure 2. The cores are described from the oldest to the youngest deposits.

#### 2.2.1. Core L14-04 and Hand-Pieces L14-04B and L14-04C

The core L14-04 has a length of 8.1 m and was drilled in a thermo terrace about 2.5 km west of the Zimov’e River mouth [48]. The core can be subdivided into two units (Figure 2): first (8.1–6.43 m depth), grey to brown ice-poor silt, faintly laminated with distinct black spots of reduced organic material and a micro lens-like cryostructure, and second (6.43–0 m), grey to brown ice-rich silt with rare spots of peaty inclusions, mostly with lens-like to blocky cryostructure [48]. A core barrel loss in the borehole prevented further drilling and four samples (hand-pieces) L14-04B were collected from the coastal bluff from layers resembling the upper part of core L14-04 while five samples L14-04C originated from ice-wedge casts resembling the lower part of the core.

#### 2.2.2. Core L14-03

The core L14-03 has a length of 15.49 m and was drilled in a thermo-terrace about 1.0 km west of the Zimov’e River mouth and extends the record of the next core (L14-02) from a lower topographic position [48]. The core can be subdivided into six units from bottom to the top (Figure 2): first (15.49–13.7 m), sand and gravel layers, which could not be sub-sampled (these stony deposits stopped further progress in the borehole), second (13.7–11.95 m), composite ice-sand wedge, ice-supported pebbles and partly clear ice, third (11.95–9.59 m), rich in vertical ice bands, fourth (9.59–8.62 m), ice-rich sand and pebble layers, fifth (8.62–6.02 m), composite ice-sand wedge, rich in vertical ice bands, and sixth (6.02–0 m), grey to brown silt with rare plant remains and lens-like cryostructure, partly with cm-thick ice bands.

#### 2.2.3. Core L14-02

The core L14-02 has a length of 20.02 m. The drilling site was located on a Yedoma hill around 800 m west of the Zimov’e River mouth. The core can be subdivided into two main units (Figure 2). The first (10.92–20.02 m) is composed of ice-wedge ice, which was not sampled for DNA analyses. The second unit (0–10.92 m) comprises Yedoma deposits composed of grey to brown and olive ice-rich silts with scattered plant remains and peaty inclusions and a mainly coarse lense-like cryotexture, partly with cm-thick ice bands. The core segment consists of a succession of palaeosol horizons.

#### 2.2.4. Core L14-05

The core L14-05 has a length of 7.89 m and was drilled about 4 km west of the Zimov’e River mouth in a thermokarst depression (alas). The core is described as one unit of grey to brown silt with scattered plant remains and thin layers of peaty material, mostly with lattice to lens-like cryostructure (Figure 2). In general, the ice-content increased towards the surface [48].

### 2.3. Radiocarbon Dating

Radiocarbon dating was performed using accelerator mass spectrometry (AMS) (6 MV Tandetron (High Voltage Engineering Europa B.V. (HVE), Amersfoort, Netherlands)) on plant macrofossils from 27 samples at the CologneAMS laboratory (University of Cologne, Germany) [53] following the same procedure as described in detail in [54]. Samples were corrected for carbon contributions from exogenous sources using size-matched, radiocarbon-free coal, which was processed similar to the samples. Samples close to the radiocarbon method limit were blank corrected only (a) if the ^14^C concentration (fraction modern—Fm) of the sample was larger than those of the corresponding process blank; and (b) if twice the error of the sample’s Fm value was smaller than the sample Fm [55]. The limiting age is calculated as −8033 × ln (2σ) because the 6 MV AMS at Cologne produces low blank values, due to its higher energy. The conventional radiocarbon ages are given in years before present (yr BP) and were calibrated (cal yr BP) in CALIB 7.0.2 using the IntCal13 calibration curve [56,57] (Table 1).

### 2.4. Core Sub-Sampling

The sub-sampling of the permafrost sediment cores took place in a windowless climate chamber without any contact to laboratories in which molecular genetics work is performed. The temperature was set to −10 °C to prevent thawing of the core material. The sub-sampling procedure was carried out following the protocol described in [30] using the same equipment, treatments and facilities. In total, 72 subsamples (~2 samples per m) were collected for both sedaDNA and palynological analyses and stored at −20 °C, and +4 °C, respectively.

### 2.5. Molecular Genetic Laboratory Work

#### 2.5.1. Sedimentary Ancient DNA Metabarcoding Approach

Sedimentary ancient DNA extractions and polymerase chain reactions (PCRs) followed the same procedures as described in [30]. In total, 72 samples and 8 extraction negative controls were processed, with extraction negative controls processed in the same way as the samples.

Generally, two positive PCR products were pooled for sequencing under the condition that the extraction and PCR negative controls remained negative. There were two exceptions. First, for one sample (core L14-03, 5.13 m depth) only one positive amplification could be retrieved. Second, for one PCR batch, which contained the deepest sample of core L14-04 and all hand-pieces, 2 out of 3 PCRs showed a PCR-product after gel-electrophoresis in the extraction negative control. Therefore, only the single PCR-products with the corresponding clean extraction negative controls were sequenced in the same run as all other samples. To identify the source taxa responsible for the PCR product of this extraction negative control, we pooled the two corresponding PCR products of the whole PCR batch (samples, extraction negative controls and PCR negative controls) and sequenced them in another sequencing run. This batch showed only exotic taxa in the extraction negative control (Musaceae, PACMAD clade, *Persea* and *Ruta graveolens*). The PCR negative controls of this batch showed no bands after the gel-electophoresis, yet 4 taxa with at least 10 sequence counts were obtained (Saliceae 724 counts, *Potentilla* 42 counts, Anthemidae 35 counts and *Puccinellia* 10 counts), which is why we decided to use the clean batch for our analyses.

We purified all PCR products using the MinElute PCR Purification Kit (Qiagen, Hilden, Germany), following the manufacturer’s recommendations. Elution was carried out twice to a final volume of 40 µL. The DNA concentrations were estimated with the double stranded (dsDNA) BR Assay and the Qubit^®^ 2.0 fluorometer (Invitrogen, Carlsbad, CA, USA) using 1 µL of the purified amplifications. Aiming for balanced DNA concentration among all samples, the purified PCR-products were pooled such that 60 ng DNA of each PCR-product was in the final multiplex. All extraction and PCR negative controls were included in the sequencing run. When their DNA concentration was below the Qubit detection limit, a standardised volume of 10 µL was added. Library preparation and paired-end sequencing were performed by Fasteris SA sequencing service (Geneva, Switzerland) using a Illumina HiSeq 2500 platform (2 × 125 bp) (Illumina Inc., San Diego, CA, USA) aiming for 10 Gb output. The library preparation using the HiSeq SBS V4 kit followed the MetaFast protocol, which has been developed by Fasteris especially for PCR amplicon metagenomic analyses, which is characterized by low diversity and low complexity of the amplicons.

#### 2.5.2. Specific Amplification of *Larix* from sedaDNA

In 12 samples we detected reads assigned to *Larix* at the genus level or to *Larix sibirica*. To validate the presence of *Larix*-derived DNA in these samples (especially in those with less than 20 sequence counts) and to assess the potential of analysing single-nucleotide polymorphisms (SNPs) in sedimentary ancient DNA we developed new genetic markers. Based on a chloroplast genome-wide comparison between 12 individuals from the range of *Larix gmelinii* and 7 from the range of *Larix cajanderi* [58] we chose two SNPs to develop *Larix*-specific primer pairs for short amplicons using Primer3 [59] (Table 2). Primer specificity was assessed with *in silico* PCR on a database created from the EMBL Nucleotide Sequence Database release 129 using *ecoPCR* [60], which is also implemented in the OBITools [61]. *EcoPCR* generates a list of all sequences, including their associated taxonomic name, which can potentially be amplified with the given primers (Table 2). We allowed for three mismatches in the primer sequence, but no mismatches in the last two bases at the 3’ end.

The PCRs were prepared in the exact same reaction composition and volume as described for *trnL* g and h primers in [30], with the exception of adding 4 µL DNA. The PCRs were carried out in the TProfessional Basic thermo cycler (Biometra GmbH, Göttingen, Germany): 94 °C for 5 min, followed by 55 cycles of 94 °C for 30 s, 58 °C for 30 s, 68 °C for 30 s and a final extension at 72 °C for 10 min. To monitor contamination the corresponding extraction negative controls were included as well as a PCR negative control. The negative controls were treated identically to the samples. The amplification success and product sizes were visually checked by gel-electrophoresis. The PCR products were purified using the MinElute Purification kit as described in Section 2.5.1. An A’ overhang was produced by incubation with Sigma Taq DNA polymerase (Sigma-Aldrich Chemie GmbH, Taufkirchen, Germany) and 0.2 mM mixed dNTPs (Qiagen, Hilden, Germany) for 10 min at 72 °C. The samples were cloned using the TOPO^®^ TA Cloning^®^ Kit (Thermo Fisher Scientific, Waltham, MA, USA) according to the manufacturer’s recommendations. For each cloned PCR-product, up to 15 clones were picked and re-amplified using T3 and T7 primers, with the exception of samples at 3.3 m depth (L14-05) and at 7.53 m (L14-02), amplified with cp77444, for which only 2 and 1 clones, respectively, could be retrieved. Re-amplification reaction contained 1U Sigma Taq, 10× Sigma buffer, 2 mM MgSO4, 0.25 mM mixed dNTPs, 0.8 mg Bovine Serum Albumin (VWR, Radnor, PA, USA), 0.2 mM of each primer and 2 µL DNA in a final volume of 20 µL and were carried out in the iCycler^®^ thermal cycler (Bio-Rad, Hercules, CA, USA): 94 °C for 5 min, followed by 35 cycles of 94 °C for 30 s, 52 °C for 30 s, 72 °C for 30 s and a final extension at 72 °C for 10 min. The clone-derived PCR-products were sequenced through Sanger sequencing at the LGC DNA sequencing services (Berlin, Germany).

The primers of the retrieved sequences were trimmed without allowing for mismatches in the forward or reverse primer and aligned using the MAFFT multiple aligner v. 1.3.3 plug-in [62] in Geneious v. 7.1.4 [63] with default settings. To check target region specificity, the trimmed sequences were mapped to the chloroplast genome reference sequences [58] and subjected to a BLASTn search [64] in the National Center for Biotechnology Information (NCBI) sequence database.

#### 2.5.3. Filtering of Illumina Sequencing Data and Taxonomic Assignments

The sequence quality was checked using FastQC [65] and all bioinformatic filtering steps were performed using OBITools [61] as described in [30]. The taxonomic assignments were performed using two reference databases as described in [66]: (1) the quality-checked and curated Arctic and Boreal vascular plant and bryophyte reference libraries [28,41,67]; and (2) the European Molecular Biology Laboratory (EMBL) Nucleotide Database standard sequence release 127 [68]. We use the term “sequence” to refer to the nucleotide sequence, which was generated during sequencing and to “sequence counts” to refer to the number of times this nucleotide sequence was generated. The sequences were assigned their taxonomic names due to sequence similarity to each of the reference databases using *ecotag*, following the NCBI taxonomy nomenclature [69]. If the query sequence is identical with exactly one database entry, the taxonomic name of the species will be assigned. However, if several database entries are identical, the query sequence will be assigned on a higher taxonomic level to the most recent common ancestor [61,70], e.g., on tribe (e.g., Saliceae) or family level. Hence, the taxonomic resolution of the sedaDNA dataset depends on the marker resolution for vascular plant taxa as well as the completeness of the reference database. When the same taxonomic name was assigned more than once, we grouped them together to mitigate a possible over-estimation of taxonomic richness.

Rare sequences that exhibited less than 10 counts in a sample were excluded from the analyses as they can arise through tag-jumps [71] and might increase the rate of false positives. Furthermore, only sequences were kept that matched 100% to an entry in a reference database. Exotic sequences assigned to cultivated plants or those highly unlikely to occur in arctic or boreal landscapes were also excluded from our analyses.

### 2.6. Pollen Sample Treatment and Analysis

In total, 72 samples were palynologically analysed. Pollen extraction of 1 mL sediment was performed following the standard procedure of [72] including HCL, KOH, HF (including boiling) and actetolysis. A *Lycopodium* spore tablet (Batch no. 1031; *n* = 20,848 ± 1460) was added to each sample to estimate the pollen concentration [73]. Pollen slides were analysed with a Zeiss Axioskop 2 light microscope (400–600× magnification). At least 300 pollen grains were identified in each sample. Taxonomic identification was based on published pollen and non-pollen palynomorph atlases [74,75,76,77,78,79,80,81,82,83,84] and a pollen reference collection at the Arctic and Antarctic Research Institute (Saint Petersburg) and the Alfred Wegener Institute (Potsdam). The systematic placement of Chenopodiaceae changed after the application of molecular techniques and is now included into Amaranthaceae [85]. To facilitate comparability with previous palynological studies in the Laptev region, we use Chenopodiaceae in this manuscript.

### 2.7. Statistical Analyses and Visualisation

For statistical analyses we excluded aquatic macrophytes, bryophytes, algae and taxa associated with wetlands and bogs, such as Cyperaceae from both datasets to reduce the effect of different depositional conditions, since we focus on changes in the terrestrial vegetation. Furthermore, we are interested in comparing the richness of terrestrial vascular plant taxa between the different time periods covered. Comparisons of taxonomic richness between samples with different count sizes can be biased as higher count sizes increase the chance of finding rare taxa. Therefore, we rarefied the data using R-functions *rarecurve* and *rarefy*. This permitted us to compare the richness at the same count size, determined by the minimum number of retrieved sequence counts among all samples (L14-03, 1.91 m = 893 counts) or by the minimum number of pollen (L14-03, 12.8 m = 82 counts) [86,87]. Two samples were excluded from the rarefaction analysis as they had very low sequence count sizes (L14-04, 2.05 m = 32 counts, L14-05, 7.22 m = 182 counts) and would have decreased the rarefied taxonomic richness far too much.

We used multivariate statistical analyses to explore the patterns of the multivariate data and to identify samples with a similar taxonomic composition from among the different cores, whose deposits comprise partially overlapping time-slices. We grouped the taxa to family levels, except for taxa with erect shrub or tree growth-form to get a better signal. If a family was represented by only one species or genus, we retained the species or genus name. Afterwards, we calculated the relative proportions of each taxon within each sample, and applied transformation by the fourth root for sedaDNA data and by square root for pollen data to mitigate the effects of very dominant and very rare taxa. We ran a principal component analysis using *rda* to assess the major structure of the multivariate dataset (see also Figures S2 and S3 in Supplement1.docx). We plotted the stratigraphic diagrams for each core separately using *strat.plot*. All statistical analyses were performed in R v. 3.0.3 [88] using the packages “vegan” [89], “rioja” [90] and “analogue” [91,92]. The original raw data are available at PANGAEA [93] and the terrestrial vascular plant datasets are available as supplementary material (Tables S1 and S5).

## 3. Results

### 3.1. Radiocarbon Ages

Twenty-seven samples were radiocarbon dated (Table 2). The lower part of core L14-03 had sediments older than 52 kyr BP, while the upper part was deposited between about 29.3 and 4 kyr BP (33.4 to 4.5 cal kyr BP). The deepest part of core L14-02 exhibited ages near the analytical capacity of the 6 MV Tandetron in Cologne until about 5.33–5.56 m depth. The upper part accumulated between 46.3 and 37.3 kyr BP, although samples between 3.47–3.77 m and 4.62–4.78 m do not age continuously with increasing depth. Such age inversions result from cryoturbations as well as from the high uncertainty range of ages near the radiocarbon dating limit [8]. The deepest unit below 5.83 m of core L14-05 returned ages of 51 to 54 kyr BP. They belong to thawed and refrozen Yedoma deposits below a thermokarst depression, called taberite. The sample at 5.00–5.17 m showed a very young age of 1.2 kyr BP, while samples from between 1.39–1.69 m and 0.65–0.85 m accumulated around 10.1 kyr BP and 2.2 kyr BP, respectively. The very young age at 5.00–5.17 m is probably due to the relocation of material during drilling. Overall, the radiocarbon ages support stratigraphical interpretations based on previous work [4,7,8,25,52,94].

### 3.2. Overall Composition of the sedaDNA Metabarcoding and Pollen Data

Illumina sequencing generated a total of 28,827,030 sequence counts. After bioinformatic filtering 11,808,836 sequence counts were assigned to 347 plant and algae sequences with a 100% match to an entry in a reference database. Of these, we assigned 5 sequences (233,081 counts) to trees, 5 (2,780,400 counts) to erect shrubs, 212 (5,200,502 counts) to herbs graminoids and non-erect dwarf-shrubs (including graminoids), 39 (1,737,207 counts) to swamp and aquatic (including sedges) taxa, 52 (123,958 counts) to bryophytes and 6 (3884 counts) to algae. Furthermore, we assigned and subsequently discarded 28 sequences (1,729,804 counts) to exotic taxa, since they are unlikely to have occurred in the Arctic and belong to cultivated and food plants. Three samples contained only exotic sequences after filtering, hence only 69 samples were further analysed.

The PCR negative controls of the sequencing run were all clean. Seven out of eight extraction negative controls were clean in the gel-electrophoresis picture while only five out of eight extractions negative controls were clean after sequencing (Table S2–S4). However, as we show in the Supplementary material (Tables S1–S4), the sequences found in those extraction negative controls do not compromise the results and interpretations of the associated samples.

The palynological analyses identified 57 terrestrial vascular plant taxa. Of these, the dominant taxa in the pollen assemblages are Poaceae and Asteraceae (especially *Artemisia*), followed by *Alnus*, *Betula* and Caryophyllaceae.

### 3.3. Temporal Changes of Trees and Shrubs

#### 3.3.1. *Sed*aDNA Metabarcoding

**Figure 3:**
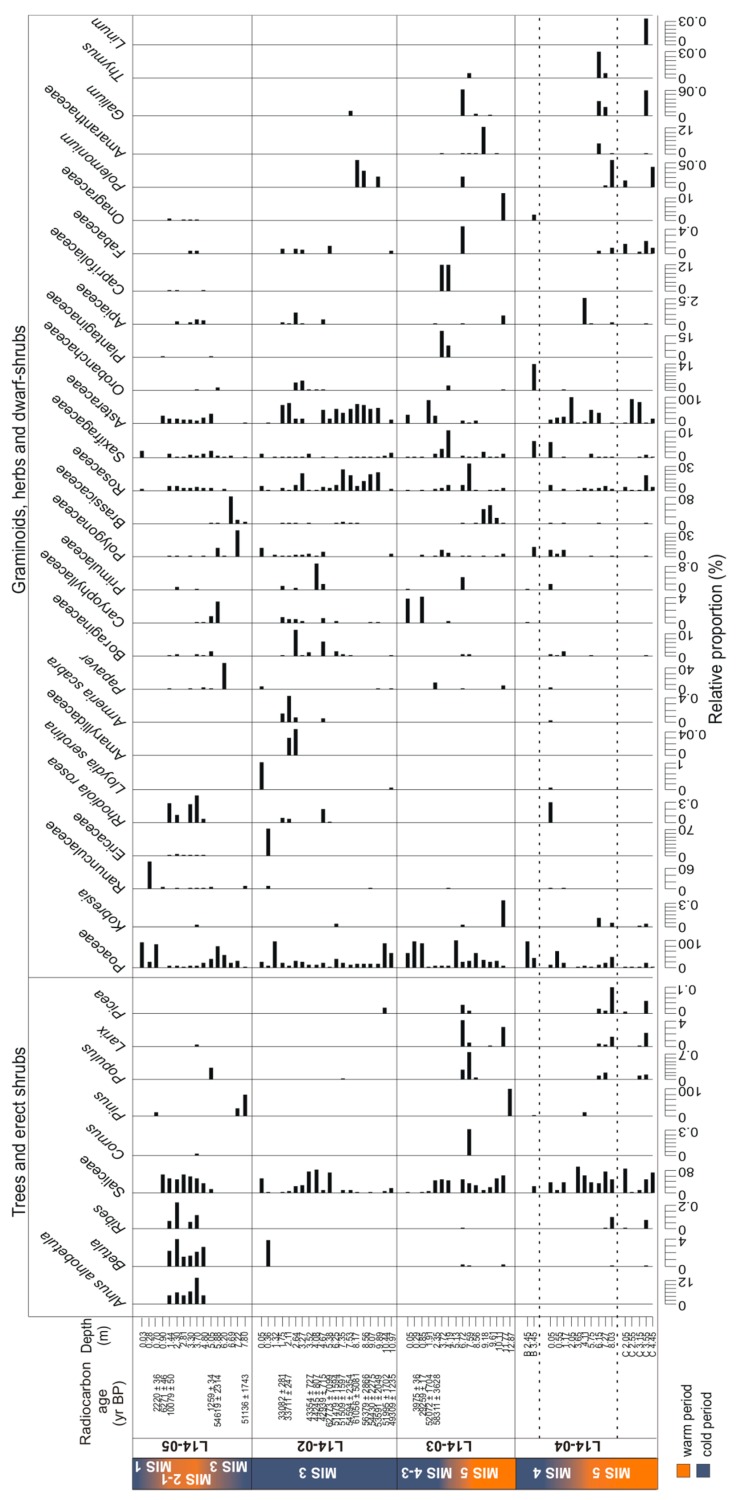
Stratigraphic diagram representing relative proportions of terrestrial sedaDNA sequences in each sample of the four permafrost cores (L14-05, L14-02, L14-03, L14-04) and the hand-pieces (collected soil nuggets from outcrops: L14-04B, L14-04C). The radiocarbon ages (yr BP) and the corresponding marine isotope stages (MIS) are displayed on the left side, with warmer periods in orange and colder periods in blue. The scales of the relative proportions are for each taxon.

**Figure 4:**
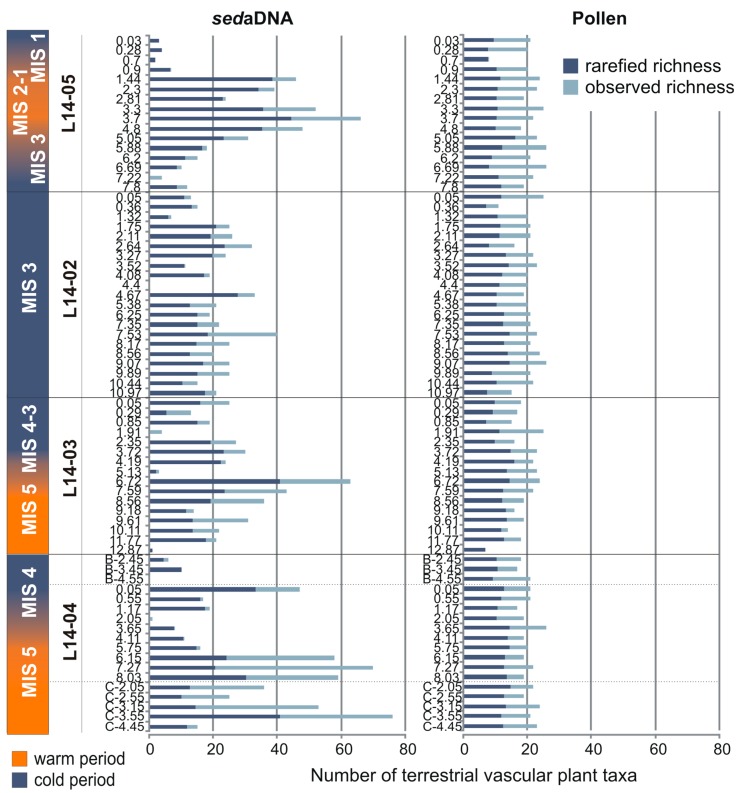
Richness of observed (light blue) and rarefied (dark blue) terrestrial vascular plant taxa for the *sedaDNA* and pollen datasets. The corresponding MIS are displayed on the left side, with warmer periods in orange and colder periods in blue.

The core L14-04 is mainly composed of sequences assigned to Saliceae, Poaceae, Asteraceae and Rosaceae (Figure 3). The rarefied richness ranges between 8 and 33 taxa (Figure 4). With increasing depth, richness first decreases strongly until 3.65 m (MIS 4) and then increases again towards high richness in the lowest part of the core between 8.03 and 6.15 m (MIS 5). This part of the core is characterised by the presence of trees (*Larix*, *Picea*, *Populus* and *Pinus*) and shrubs (*Alnus*, *Betula*, *Ribes*, *Saliceae* and *Cornus*) as well as Ericaceae, *Galium* and *Kobresia*, which suggests a warm period. Above 6.15 m no tree or shrub taxa are present, except for *Pinus* at 4.11 m and Saliceae.

The hand-pieces of L14-04B (putatively marine isotope stage 4 (MIS 4)) should resemble the upper part of core L14-04, but this is not the case. Rarefied richness varies between 4 and 10 taxa. The sample at 3.45 m is mainly composed of Poaceae, Saliceae, *Pedicularis*, Polygonaceae, Saxifragaceae, Onagraceae and *Pinus*. The sample at 2.45 m is mainly composed of Poaceae, accompanied by Asteraceae, Caryophyllaceae and Primulaceae. Both samples display a composition which does not match any other sample of core L14-04, but show some similarities to samples from core L14-03.

The five hand-pieces of L14-04C (MIS 5) are very different in their taxonomic composition and were collected to extend the record of core L14-04. The taxonomic richness ranges between 10 and 14 taxa, although the sample at 3.55 m is composed of 41 taxa. Similar to the lowest part of L14-04, the samples comprise trees (*Larix*, *Picea*, *Populus, Pinus*) and shrubs (*Alnus*, *Betula*, *Ribes, Saliceae*) with some Ericaceae, *Galium* and *Kobresia*, but not in all samples. The only woody taxon present in the deepest sample at 4.45 m is Saliceae.

In core L14-03, rarefied richness ranges between 1 and 41 taxa. The deepest sample at 12.87 m comprises only *Pinus* sequences and probably reflects the poor DNA preservation conditions of the pebbly sediments. Tree and shrub taxa are present between 11.77 m and 6.72 m, except for samples at 10.11 and 9.18 m (MIS 5). At 11.77 m, *Larix* is only accompanied by *Betula* and Saliceae, while samples at 7.59 and 6.72 m display a similar taxonomic composition to the deepest part of core L14-04 and the hand-pieces L14-04C (MIS 5). In these samples, trees include *Larix*, *Picea* and *Populus* and shrubs include *Alnus*, *Betula*, Saliceae, *Cornus* and *Ribes*, while herbs and non-erect dwarf-shrubs include, amongst others, *Kobresia*, Ericaceae and *Galium*. In the upper part of the core above 6.72 m (MIS 4-3) no tree or shrub taxa are recorded except for Saliceae. This part is characterised by a high proportion of herbs.

The rarefied richness of core L14-02 (MIS 3) ranges between 6 and 27 taxa. The number of taxa is relatively constant throughout the core except for the uppermost three samples (1.32 to 0.05 m), where it is lower. The deepest two samples contain *Picea* (10.44 m) or *Pinus* (9.89 m) but no other tree or shrub taxa apart from Saliceae. Shrubs are present in samples at 7.53 and 6.25 m (*Alnus*, *Betula,* Saliceae). The sample at 7.53 m contains *Populus*; however rare *Larix* sequence counts (<10 counts) were present in the raw dataset at 7.53 and 7.35 m, but filtered out as putative false positives. Samples in the upper part of the core are characterised by the absence of trees and shrubs except for Saliceae. The sample at 0.36 m is characterised by the presence of *Alnus*, *Betula* and Saliceae and high proportions of Ericaceae.

The taxonomic richness of core L14-05 increases with decreasing depth until 3.7 m and remains high to medium until 1.44 m (transition from MIS 2 to MIS 1). Then the richness drops sharply at 0.9 m and remains low until the uppermost sample (MIS 1). The deepest part of the core is characterised by the absence of Saliceae sequences, which first occur at 5.05 m—the sample in which *Populus* is the only tree taxon. Samples between 4.8 and 1.44 m are characterised by the presence of shrubs (*Alnus*, *Betula*, *Ribes*, *Cornus,* Saliceae) and Ericaceae. *Larix* is present at 3.7 m, but we note that in the sample at 3.3 m *Larix* was recorded with 8 read counts but discarded during the filtering process as a putative false positive. *Pinus* sequences are recorded in the two deepest samples as well as the one at 0.7 m, but no other tree or shrub taxa are present in these samples.

**Figure 5:**
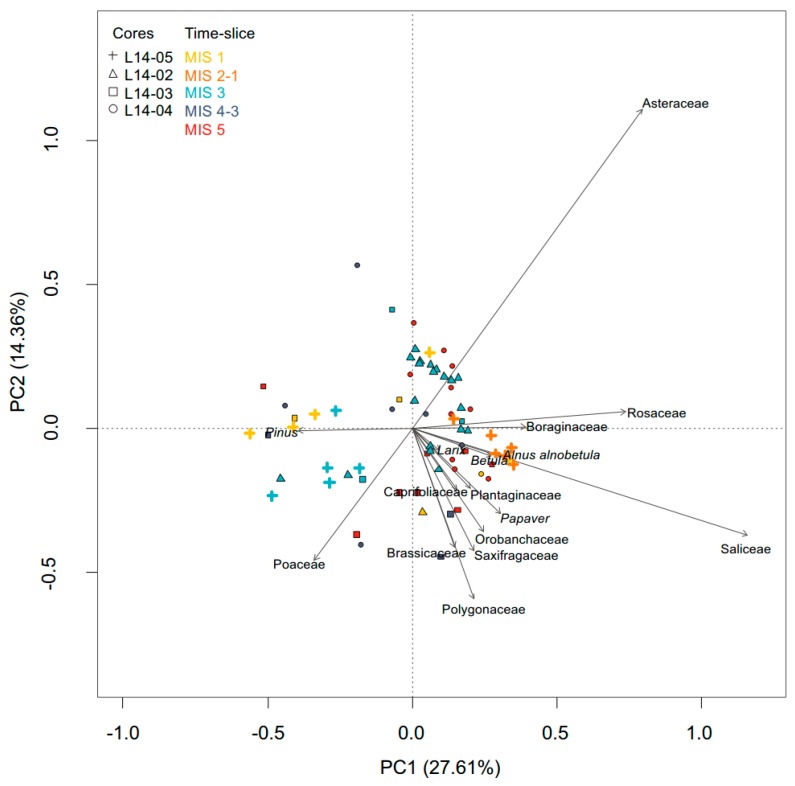
Principal Components Analysis (PCA) biplot comprising the 15-principal terrestrial sedaDNA taxa (with herbs and non-erect dwarf-shrubs at family level) and *Larix*. Samples are coloured according to the corresponding marine isotope stage (MIS 1 in yellow, MIS 2 to MIS 1 transition in orange, MIS 3 in light blue, MIS 4 to MIS 3 in dark blue and MIS 5 in red) and the different symbols indicate the core from which the samples were taken (L14-05 with a cross, L14-02 a triangle, L14-03 a quadrate and L14-04 a circle).

The principal component analysis displays the major structure of the terrestrial vascular plant composition for the 69 samples processed by sedaDNA metabarcoding (Figure 5). A high proportion of the variance (41.97%) in the multivariate dataset is explained by the first two axes, with PC1 explaining 28% and PC2 14% of the variance. The variance along PC1 and PC2 can mostly be explained by Asteraceae, Saliceae, Rosaceae, Poaceae and Polygonaceae, which have the highest species scores. Except for *Pinus*, the vectors of all tree and shrub taxa point towards the lower right quadrant, along with taxa that are associated with subarctic/boreal vegetation such as *Galium* or *Cornus*. Samples containing such taxa, display positive scores on PC1, except for one sample (L14-03, 9.61 m). *Pinus*, on the other hand, is highly correlated with PC1 and its vector points towards the left quadrants in the ordination plot. Samples containing taxa that indicate open tundra vegetation with a complete absence of shrubs display negative scores on PC1. But those indicating open tundra vegetation with the presence of shrubs exhibit positive values on PC1 and are displayed mostly in the upper right quadrant of the ordination plot. Most of the samples were associated with MIS 5, the transitions from MIS 2 to MIS 1 and MIS 1 are displayed on the right side of the plot.

#### 3.3.2. Pollen

**Figure 6:**
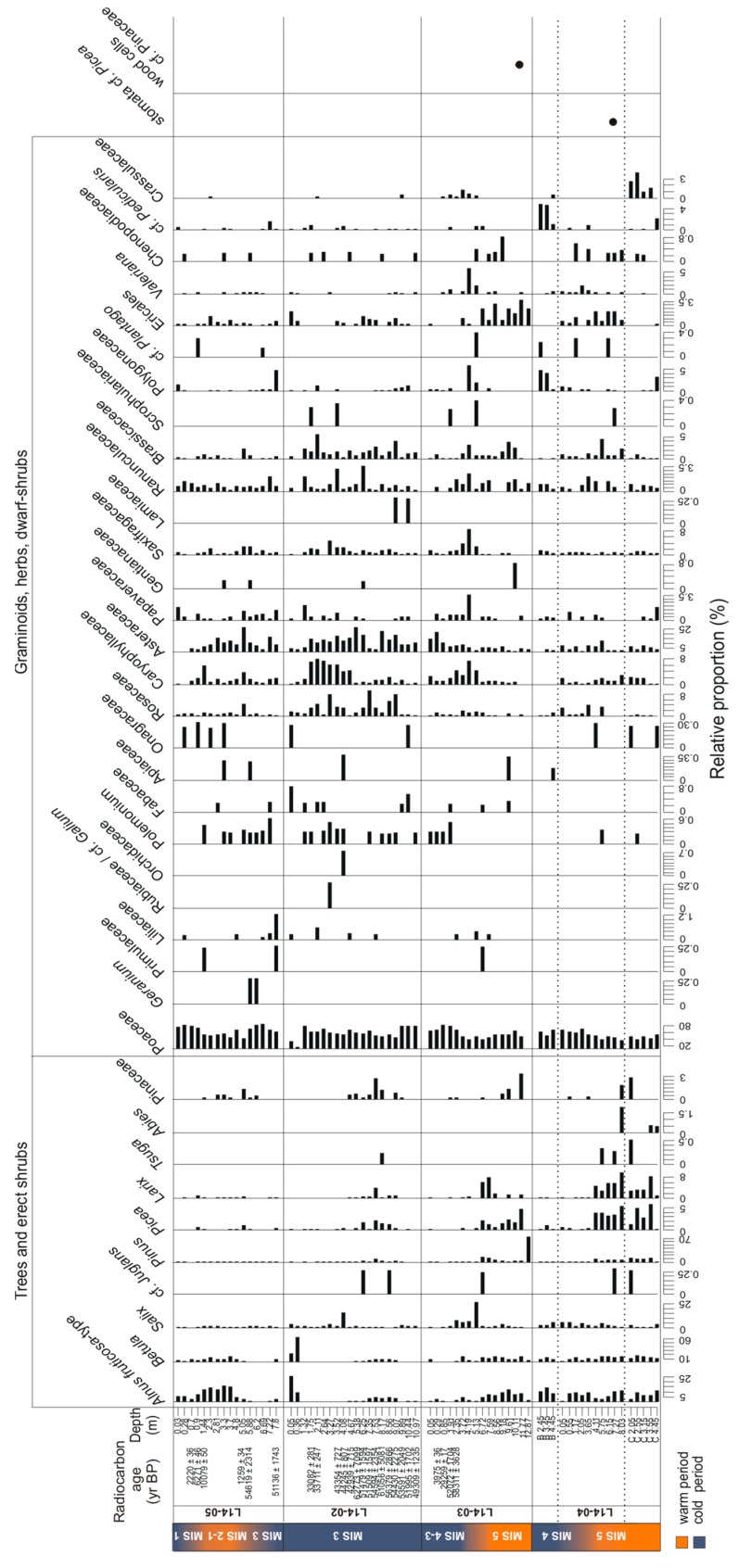
Stratigraphic diagram of relative proportions of terrestrial pollen in each sample of the four permafrost cores (L14-05, L14-02, L14-03, L14-04) and the hand-pieces (collected soil nuggets from outcrops: L14-04B, L14-04C). The presence of coniferous stomata and wood cells is marked by a black dot. The radiocarbon ages (yr BP) and the corresponding MIS are displayed on the left side, with warmer periods in orange and colder periods in blue. The scales of the relative proportions are adjusted for each taxon.

The rarefied richness of terrestrial vascular plant taxa varies between 7 and 16 and remains relatively constant in comparison to the richness displayed by the sedaDNA data (Figure 4). The palynological data show relatively high proportions of trees and shrubs throughout core L14-04 and in the hand-pieces L14-04C (MIS 5). This matches well with the sedaDNA dataset. The proportions of trees and shrubs decrease from the bottom of the core until 3.65 m (MIS 5) and remain approximately stable towards the uppermost sample (MIS 4). While *Salix* has the highest proportions in the two uppermost samples, *Alnus* peaks in the two deepest samples and *Betula* is represented in most samples at more than 10%. The proportions of *Larix*, *Picea* and *Pinus* are highest between 4.11 and 8.03 m (MIS 5), as well as of Ericales. The presence of *Picea* in the sample at 6.15 m is supported by finds of stomata cf. *Picea* in this sample (Figure 6). The highest proportion of *Pinus* is found in the sample at 4.11 m, where it is also present in the sedaDNA dataset. In the hand-pieces, the proportions of tree taxa are low for samples from L14-04B (putatively MIS 4) but relatively high for samples from L14-04C (MIS 5). *Pinus* peaks at 2.05 m (L14-04C). The deepest sample of the core at 3.55 m and the hand-piece at 4.45 m (L14-04C) contain the only records of *Abies* among all samples. A notable difference between the two datasets is the presence of *Populus* and *Ribes* in the sedaDNA dataset, which are both absent in the pollen data.

The terrestrial pollen assemblage of core L14-03 exhibits high proportions of tree and shrub taxa at 6.72, 7.59 and 11.77 m (MIS 5). Wood cells determined to the Pinaceae family support the presence of conifers in sample 11.77 m (Figure 6). Furthermore, a peak of *Pinus* pollen is found at 12.87 m. This pattern is similar to the sedaDNA dataset. The major differences between the two datasets are first, the absence of *Populus*, *Cornus* and *Ribes* in the pollen record; second, the highest proportion of *Salix* pollen is found at 5.13 m, while no Saliceae is present in the sedaDNA record; and third, *Pinus sedaDNA* is only recorded in the deepest sample, while it is absent at the level where it has the highest proportion in the pollen record.

In core L14-02 (MIS 3) the proportions of tree and shrub taxa in the pollen record are low. Shrub taxa are represented throughout the core and increase in the two uppermost samples (MIS 1), similar to the sedaDNA record. The proportions of trees peak at 7.53 m, with *Larix* being present between 9.07 and 4.67 m. This supports the presence of *Larix* in the sedaDNA record at 7.53 and 7.35 m as being real, although the sequences were discarded as potential false positives. *Picea* and *Pinus* are present in most of the samples at proportions of less than 1%, except for samples between 9.07 and 6.25 m, which show increased proportions but less than 3% for *Picea* and less than 10% for *Pinus*.

In core L14-05, the highest proportions of tree and shrub pollen are recorded between 5.05 and 1.44 m (transition from MIS 2 to MIS 1). A distinct difference is the presence of *Salix* pollen below 5.05 m (MIS 3), whereas Saliceae sequences are absent in the sedaDNA dataset. Furthermore, *Populus*, *Cornus* and *Ribes* are absent in the pollen record. *Alnus* and *Betula* show increased proportions between 3.7 and 1.44 m in comparison to the rest of the core. *Larix* pollen is present in most samples of this core, but at low proportions. Few indications of *Picea* and *Pinus* are recorded, with low proportions of less than 1.1%.

**Figure 7:**
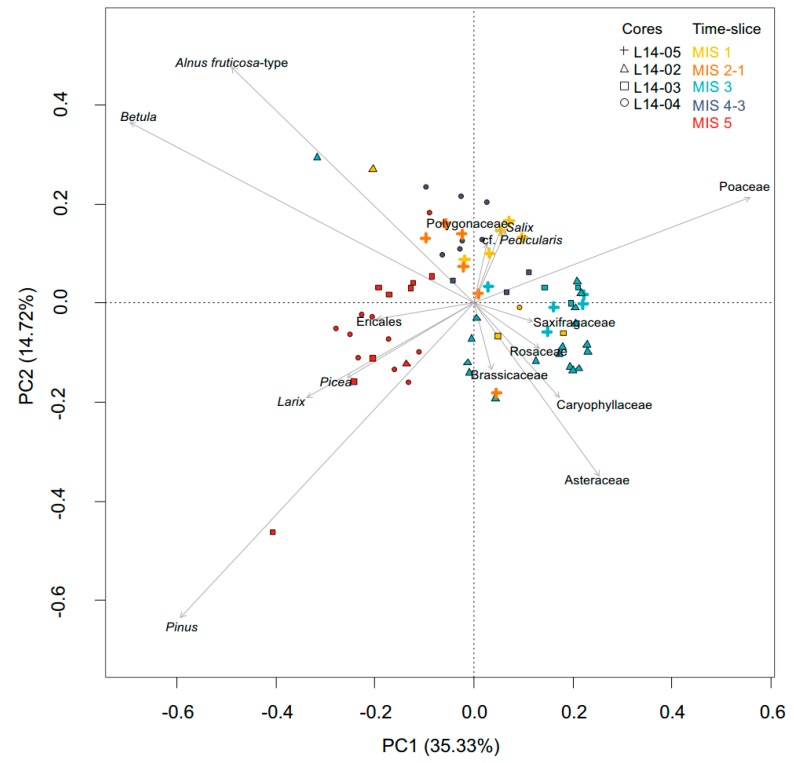
PCA biplot comprising the 15 principal terrestrial pollen taxa (with trees and shrubs pooled to genus level and with herbs and non-erect dwarf-shrubs pooled to family level) and samples. Samples are coloured according to the corresponding marine isotope stage (MIS 1 in yellow, MIS 2 to MIS 1 transition in orange, MIS 3 in light blue, MIS 4 to MIS 3 in dark blue and MIS 5 in red) and the different symbols indicate the core from which the samples were taken (L14-05 with a cross, L14-02 a triangle, L14-03 a quadrate and L14-04 a circle).

For the terrestrial pollen dataset, 35% of the variance is explained by PC1 and 15% by PC2. The highest species scores along axes PC1 and PC2 can be found for *Betula*, *Pinus*, *Alnus*, Poaceae and Asteraceae. While the vectors of taxa indicating open tundra vegetation point to the upper and lower right quadrants, those of shrub taxa point towards the upper left quadrant and those of tree taxa into the lower left quadrant (Figure 7). Samples associated to MIS 5 are displayed on the left side of the PCA biplot, mostly in the lower left quadrant. The samples associated to the transition between MIS 2 to MIS 1 and MIS 1 are mostly located in the upper left and right quadrants, together with samples associated with MIS 4 to MIS 3.

### 3.4. Validation of Sediment-Derived Larix Sequences and First Insights into Past Genetic Variation

EcoPCR results confirmed that the developed primer pairs are *Larix* specific. *In silico* PCR using primers cp77444 retrieved 3 hits, with only *Larix* showing no mismatches in both primer sequences (Table 2). The in silico PCR using the second primer pair cp103595 resulted in 14 hits comprising all genera within the Pinaceae family. Except for *Larix* all other hits have at least 2 mismatches in the reverse primer sequence. Additionally, a BLASTn search of the target sequence for both primer pairs confirmed the *Larix* specificity. Based on the current status of the NCBI database, this allows for the validation of *Larix*-derived sedaDNA in our samples. Even though *Larix* was filtered out due to a low number of sequence counts at 3.3 m depth in core L14-05, it was successfully re-amplified and re-sequenced by both markers.

High-throughput sequencing allowed the detection of *Larix* sequences in 12 samples from three of the cores (L14-03, L14-04 and L14-05) and three hand-pieces (L14-04C). Out of these, 10 samples were successfully re-amplified by the marker cp77444 and 11 samples by the marker cp103595 (Table 3). Cloning and Sanger re-sequencing was successful for 9 samples amplified by cp77444 and for 10 samples amplified by cp103595 (Table 3). All sequences aligned specifically with the target regions on the *Larix gmelinii* and *Larix cajanderi* chloroplast reference genomes and showed no mismatches in any of the primer sequences.

The first marker, cp77444, contains a SNP (A/C) at position 12 of the sequence. At this position all samples showed variant A, except for the sample at 8.03 m in core L14-04, which comprises variants of both A and C. The second marker, cp103595, contains a SNP (T/C) at the second-to-last position. In two samples only variant C was retrieved, in four samples only variant T and in four samples both variants were retrieved (Table 3).

## 4. Discussion

### 4.1. Geochronology

The association between stratigraphic units and the approximate period of deposition is based on a combination of AMS radiocarbon dating of 27 samples with pre-existing dating results and palaeoenvironmental interpretations of local outcrop sections [4,7,8,25,52,94]. In permafrost landscapes, warm interglacial conditions lead to the melting of ground ice resulting in ground subsidence (thermokarst) and lake development [95]. The core L14-04 was taken from the Krest Yuriakh Suite (MIS 5), which is located in a stratigraphical context below deposits of the Yedoma Ice Complex (MIS 3). The core contains lacustrine deposits suggesting deposition in a thermokarst lake and its lower part can be assigned to MIS 5 [7,48]. As the suggested age of the core exceeds the limits of the AMS radiocarbon method, no samples were dated with this method. However, two infrared stimulated luminescence (IRSL) dates from the adjacent outcrop [25] suggest an MIS 5 age.

According to its stratigraphy, core L14-03 was taken in a thermo-terrace and extends the record of the core L14-02 from a lower topographic position. It contains deposits of the Kuchchugui suite associated to MIS 4. The radiocarbon ages within the upper 2 m of the core suggest deposition during MIS 3 while the uppermost sample suggests deposition during MIS 1.

The core L14-02 was drilled from the ground surface at the top of a Yedoma hill. Palaeoenvironmental interpretations [8] suggest interstadial deposition during MIS 3 with a succession of palaeosols in an ice-wedge polygon. Most of the radiocarbon-dated samples exhibit ages also near the analytical capacity of the 6 MV Tandetron in Cologne. This is often observed for deposits of MIS 3 origin, leading to high uncertainties or infinite ages [8].

Due to erosion and thermokarst processes, MIS 1 deposits are mostly preserved as lake deposits in thermokarst depressions, boggy deposits after lake drainage or as cover deposits on Yedoma hills [7]. According to stratigraphic interpretations from the adjacent outcrop section [7], the sediments below 5.5 m of core L14-05 are thawed and refrozen taberite deposits of the Yedoma Ice Complex (MIS 3). Between 0.9 m and 5.5 m, the core contains lacustrine deposits, which likely formed during the late glacial (MIS 2) or the transition from the late glacial to the early Holocene. (MIS 2 to MIS 1). Above 0.9 m, there are MIS 1 sub-aeolian deposits from a boggy environment that formed after lake drainage [7].

### 4.2. Assessment of Tree and Shrub Taxa in the sedaDNA and Pollen Records

Our vascular plant derived sedimentary ancient DNA record contains tree (*Larix*, *Picea*, *Populus, Pinus*) and erect shrub taxa (*Saliceae*, *Alnus*, *Betula*, *Ribes*, *Cornus*) in several strata, which can be associated to MIS 5, the transition between MIS 2 to MIS 1 and MIS 1. Generally, the presence of trees is associated with the presence of shrubs other than Saliceae, as well as with higher taxonomic richness among terrestrial taxa. We detected *Larix* sequences in three different cores and three hand-pieces (large soil nuggets), which were collected from four localities. When *Larix* pollen proportions are more than 4%, *Larix* is also represented in the sedaDNA record. Conversely, for 5 out of 16 occurrences of *Larix* in the sedaDNA record, the pollen proportion is less than 2%, including one sample where *Larix* pollen is absent. Additionally, some samples have *Larix* pollen proportions of up to 3%, but our criteria for sequence counts to be included in the sedaDNA analysis were not met. We were able to validate the presence of *Larix*-derived sedaDNA by re-amplification and re-sequencing with different primer pairs, corroborating that the low sequence counts were not an artefact. This is in contrast to previous results based on macrofossil and pollen analyses from outcrop sections at the same sampling locations. Kienast et al. [6,26] did not find any *Larix* macro-remains on Bol’shoy Layakhovsky Island, but did find some at the Oyogos Yar coast across the Dmitry Laptev Strait, leading them to suggest that the *Larix* treeline was located in the Dmitry Laptev Strait during MIS 5. Andreev et al. [25] detected *Larix* pollen with proportions of less than 1%, which they interpreted as re-worked pollen in the putative MIS 5 sequence R22+60 (Figure 2). Hence, they conclude that *Larix* was not present at Bol’shoy Lyakhovsky at that time and that the island was covered by tundra dominated by Poaceae and *Artemisia* at the beginning of MIS 5 and by shrub tundra including *Alnus*, *Betula* and *Salix* at more protected sites during the MIS 5 optimum. However, a recent study [94] analysed pollen records from the Buchchagy Ice Complex (MIS 5) on Bol’shoy Lyakhovsky Island and detected well-preserved *Larix* pollen and *Larix*-type stomata, which supports our findings. As *Larix* pollen is generally under-represented in pollen records due to low pollen productivity and low pollen dispersal capacity [37,96], even a single pollen grain is usually accepted as evidence for the local to regional presence of *Larix* [97]. Further evidence, that *Larix* has the potential to grow at such high latitudes and even farther north was provided recently [98] by *Larix* wood from Kotelny Island (New Siberian Archipelago) dated to ~38 kyr BP (MIS 3 interstadial). However, in the corresponding time-slice of core L14-02 no *Larix* sequences were detected.

Overall, *Picea* sequences are congruent with the presence of other tree taxa. In the deepest part of core L14-02 the sedaDNA record contains *Picea* sequences, but other taxa imply an open tundra landscape, in which *Picea* was not likely to be a component of the regional species pool.

*Populus* is mostly present in samples in which other tree taxa are present, except for one sample (L14-05, 5.05 m). Further evidence of *Populus* is needed to validate its actual occurrence in the Siberian high Arctic during MIS 5. *Populus* pollen is generally under-represented in the pollen record in comparison with its abundance in the vegetation [99] and due to its very low preservation potential as it is easily destroyed [100].

While the vectors of *Larix*, *Picea* and *Populus* point towards the lower right quadrant of the ordination plot (Figure 3) along with shrubs, the *Pinus* vector is directed towards samples of low taxonomic richness, which are dominated by Poaceae, most likely reflecting sparse grass-tundra under colder climatic conditions. The presence of *Pinus* in the sedaDNA record conforms, in several samples, with the pollen data and thus could have resulted from long-distance transported pollen. As *Pinus* has a much higher pollen productivity and a much lower pollen fall speed in comparison to *Picea* and *Larix* [96,101], *Pinus* is known to display notable proportions in the pollen spectra of treeless environments such as the Arctic tundra [35,36]. The contribution of DNA from pollen to the sedaDNA record was also considered in [31]. However, it has been reported that DNA from pollen is not easily amplified [43,102,103]. In samples without a notable proportion of *Pinus* pollen, contamination could be a possible explanation, although all associated negative controls did not contain *Pinus*. The occurrence of *Pinus* in the sedaDNA record is dubious and hence not deemed an authentic part of the regional vegetation.

We consider Saliceae, *Alnus*, *Betula*, *Ribes* and *Cornus* as shrub taxa, but *Alnus*, *Betula* and *Cornus* can also take a tree growth-form. The genus *Cornus* contains species (e.g., *Cornus suecica*) that even exhibit the herb growth-form. The genera *Ribes* and *Cornus* were, however, not represented in the pollen record. Both genera are characterized by insect pollination [104,105], which usually results in an under-representation in comparison to wind-pollinated taxa. Saliceae is over-represented in the whole dataset, as was noted in a recent study [30] possibly because of the massive below-ground biomass of *Salix* dwarf-shrubs in tundra environments [106]. The short *trn*L (UAA) intron marker does not contain sufficient variation to allow the distinction between most species of *Salix* and *Populus*, leaving the resolution at tribe level (Saliceae). During colder periods, we interpret the presence of Saliceae as *Salix*, while during warmer phases, we cannot rule out a contribution of *Populus*.

Macrofossil evidence from the Oyogos Yar coast suggests that the past forest community during MIS 5 differs in comparison to the present-day northern larch forests in that they contained tree birch and alder, with birches probably dominating the vegetation [6]. As we still do not know much about the vegetation during MIS 5, we believe it could be possible that *Populus* was a component of the past vegetation mosaic, along with larch, spruce, birch and alder.

### 4.3. Terrestrial Plant Community Changes of Warm Phases since the Last Interglacial

Generally, samples reflecting warm phases display a high taxonomic richness of terrestrial vascular plants and are characterised by the presence of trees and shrubs in both the sedaDNA and the pollen records. Samples from the lower part of core L14-04 and the hand-pieces L14-04C are among the most diverse samples recorded in this study and comprise the tree taxa *Larix*, *Picea* and *Populus*, shrub taxa such as *Ribes*, *Cornus*, *Alnus* and *Betula*, as well as herbs such as *Galium* and Ericaceae. The sedaDNA and pollen records align well within the stratigraphic units. Core L14-03 contains a warm phase older than 58.3 ± 3.6 ^14^C kyr BP, starting at 11.77 m and ending approximately at 6.72 m in both the pollen and the sedaDNA data. The associated samples intermix with those from the core L14-04 and the hand-pieces L14-04C in the PCA ordination plot. Hence, an MIS 5 origin for these samples can be confirmed. The vegetation was likely a mosaic composed of open *Larix*, *Picea* and *Populus* forest, accompanied by erect (dwarf-) shrubs such as *Alnus*, *Betula*, *Salix*, *Cornus* and *Ribes*, as well as Ericaceae and *Galium* in the understorey. There were probably also open spots with *Kobresia*, a typical steppe taxon, and *Papaver*, a typical pioneer plant [6].

When the last interglacial ended, the cool Weichselian glacial period began and prompted pronounced vegetation responses. Our record shows reduced diversity in MIS 4 and an absence of trees and shrubs (except for Saliceae), which indicates at least a strong fragmentation or a general treeline retreat. Subsequently, the warmer MIS 3 interstadial shows intermediate taxonomic richness, at a level comparable with the MIS 4 period. The vegetation was open and dominated by herbs.

The Yedoma Ice Complex core L14-02 contains two warm phases. The palynological data suggest a first warm phase between 8.56 m and 5.38 m in which radiocarbon dating suggests was deposited during the early MIS 3 interstadial. This part contains very rare sequences of *Larix* at 7.35 and 7.53 m depth, which were discarded during the filtering process as probable artefacts. However, we were able to re-amplify *Larix* from a sample at 7.53 m depth with a different *Larix*-specific marker, corroborating the actual presence of *Larix*-DNA in the sedimentary record. Additionally, these two samples contain *Populus* (7.35 m) or *Alnus* and *Galium* (7.53 m) sequences. In comparison to the samples attributed to MIS 5, these samples show a much lower taxonomic richness and Ericaceae, *Picea*, *Ribes* or *Cornus* are not detected. The second warm phase is captured by both proxies in the near-surface samples at 0.36 m and 0.05 m and post-dates the Last Glacial Maximum (LGM). It is interpreted to be of Holocene origin and is characterised by Ericaceae/Ericales, *Alnus* and *Betula*, but it does not record any tree taxa, except for low proportions of *Picea* pollen.

The thermokarst depression deposits of core L14-05, especially between 3.7 m and 1.44 m reflect a warm phase during the transition from MIS 2 to MIS 1 and early MIS 1. On Bol’shoy Lyakhovsky Island, this time interval displays high taxonomic richness and comprises *Larix* as well as *Betula*, *Alnus*, *Ribes* and *Cornus* in addition to Saliceae. The presence of *Larix* on Bol’shoy Lyakhovsky Island after the LGM is remarkable, and gives weight to the current hypothesis that *Larix* persisted at high latitudes during the LGM [107,108] on sites with a favourable microclimate [109].

Over the course of the MIS 1, the record shows a decrease in diversity. The vegetation changes into high arctic treeless tundra with Saliceae as the only shrub taxon in the sedaDNA data. This supports the findings of a pollen record that displayed shrub associations at Bol’shoy Lyakhovsky in the early MIS 1 with a gradual disappearance of shrubs after approximately 7.6 ^14^C kyr BP [4].

Today, Bol’shoy Lyakhovsky is treeless and the question is, at which point in time did *Larix* disappear and why? Our sedaDNA data indicate the presence of *Larix* after the LGM, putatively during the Bølling-Allerød interstadial complex; yet *Larix* pollen is recorded in the late Holocene, in samples younger than about 2000 years. A possible explanation for the disappearance of larch could be the strong change in environmental conditions from a continental to a maritime setting caused by the MIS 1 marine transgression. In contrast to continental conditions, which are characterised by strong differences in seasonality with very cold winters and warm summers, maritime conditions buffer such temperature differences leading to relatively cool summers and milder winters [110]. Cooler summers could have decreased local evaporation rates leading to moister conditions [6,111]. As larch is adapted to extreme continentality, they are especially prone to water stress. Near Yakutsk, for example, a strong increase in precipitation in two consecutive years resulted in anoxic conditions in the rooting zone, leading at first to premature withering, and subsequently to the dieback of *Larix cajanderi* trees in the third year [112].

The present abiotic conditions in the Laptev Region are not comparable to those of the last interglacial. Based on macrofossil analyses Kienast et al. [6,26] suggested continental climate conditions due to tectonic subsidence leading to a putatively a less inundated Laptev Sea Shelf, which could have led in combination with higher solar insolation during summer to a longer growing season and drier soils.

Climate-vegetation models project an advance of the treeline in northern Siberia, reaching approximately to the coast by AD 2100, as well as a dominance of evergreen conifers [18]. Hence, MIS 5 could eventually provide a putative analogue for the future vegetation of northern Siberia under ongoing climate warming. The northwards spread of trees into areas which are presently covered by tundra and of evergreen *Picea* into deciduous *Larix* taiga would strongly affect the snow cover and albedo at high latitudes, leading to a positive climate feedback [22]; something which should be considered in future climate models. Yet, time-lags [24] due to, for example, the long generation time of conifers or competition, probably restrict the speed of a general treeline advance and the northward shift of *Picea*, making the modelled scenario of a coastal treeline by AD 2100 unlikely.

### 4.4. Past Genetic Diversity of Larch Populations on Bol’shoy Lyakhovsky Island

Initial attempts at analysing the past genetic diversity of larches [108] successfully analysed ancient DNA from 2000 to 7000 year-old larch macrofossils and a 16,000 year old larch twig from a mammoth intestine. By comparing the mitochondrial haplotypes between these macrofossils with the distribution of haplotypes in modern populations, it was concluded that admixed *Larix sibirica* × *Larix sukacewii* populations were already present on the southern Yamal Peninsula during the early Holocene [108]. This demonstrates that knowledge about the past distribution of genetic variation can improve our phylogeographic understanding of *Larix*. However, retrieving and analysing genetic variation from sediments is challenging as the primer pairs used to amplify the target region have to be specific for the taxon in question. The second objective of this paper was therefore to assess the potential of two newly designed paternally-inherited chloroplast markers, cp77444 and cp103595, to detect past genetic diversity from ancient sediments.

Overall, the amplification and cloning success is highest in samples of MIS 5 origin. As higher read counts are detected in these samples compared to some younger ones, we think that the amount of *Larix*-derived DNA is higher in these samples, increasing the chance of amplifying the target sequence. Regarding marker cp77444, most samples show only variant A, while the sample at 8.03 m depth of core L14-04 shows two variants. This implies that during MIS 5 both variants were present at Bol’shoy Lyakhovsky. Today, the prevailing larch species on the Siberian mainland south of Bol’shoy Lyakhovsky is *Larix cajanderi* Mayr, which covers a vast area (~120–160° E [113]) between the Lena River in the west and the easternmost parts of Siberia in the Chukotka region. Adjacent to *Larix cajanderi*, *Larix gmelinii* (Rupr.) Rupr. covers an area (~90–120° E [113]) from the Lena River in the east to the southern Taymyr Peninsula in the west. In a chloroplast genome-wide analysis of 19 individuals from across the tundra-taiga ecotone covering the ranges of *Larix gmelinii* (12 individuals) and of *Larix cajanderi* (7 individuals) a variant C of marker cp77444 was found, but only in individuals from the range of *Larix gmelinii* (southern Taymyr region, north-central Siberia), while individuals from the range of *Larix cajanderi* (lower Omoloy and lower Kolyma regions, north-eastern Siberia) exhibited variant A [58]. This suggests that variant A is probably not only dominant in the present but was also dominant in the past. The presence of variant C on Bol’shoy Lyakhovsky Island, which was only detected in individuals from the southern Taymyr region [87], indicates that this variant was, however, not as restricted geographically in the past as it probably is today.

Similar to the first marker, cp103595 displays both possible variants in the samples attributed to MIS 5 from core L14-04, implying that both variants were present at Bol’shoy Lyakhovsky during that time. Both variants of the cp103595 marker are distributed across both species ranges [58].

## 5. Conclusions

In contrast to previous pollen and macrofossil studies, which have reconstructed shrub-tundra as the prevailing vegetation on Bol’shoy Lyakhovsky Island, our proxies of pollen and sedaDNA indicate that trees were present during MIS 5 and during the transition from MIS 2 to MIS 1. Furthermore, the sedaDNA record suggests that Siberian northern forests were more diverse during the last interglacial than they are today. In comparison to the contemporary monodominant *Larix* forest, forests of MIS 5 additionally contained evergreen *Picea* and deciduous *Populus*. *Picea* and *Populus* likely underwent a range contraction, retreating southward, due to climate cooling after MIS 5 and only *Larix* was present afterwards. According to the pollen data, *Larix, Alnus* and *Betula* gradually disappeared over the course of MIS 1, which was probably the result of the marine transgression and the associated change from continental to oceanic conditions.

We successfully validated the presence of *Larix* in the sedaDNA using two novel chloroplast markers and were able to detect two possible variants for each SNP in one MIS 5 sample. For one marker we found that variant C, which has so far only been detected in the Southern Taymyr region among modern populations, was distributed across a much larger area in the past than it is today. The novel markers are suitable for amplicon sequencing of ancient and modern *Larix*-DNA from environmental samples such as peat, lake or permafrost sediments.

## Figures and Tables

**Table 1:** Accelerator mass spectrometry (AMS) radiocarbon ages (years before present, yr BP) and calibrated ages (cal yr BP) based on the IntCal13 calibration curve (2-sigma). Ages exceeding the limit for AMS radiocarbon dating were not calibrated (n.c: not calculable).

Core	Depth (m)	Lab Code	Plant Remains	Radiocarbon Age (Yr BP)	± (Yr BP)	Calibrated Age (Cal Yr BP)	± (Yr BP)
L14-05	0.65–0.85	COL3744.1.1	Poales, Amblystegiaceae	2220	36	2239.5	91
L14-05	0.85–1.04	COL3745.1.1	Poales, Amblystegiaceae (*Drepanocladus*, *Campylium*)	6271	46	7221	70
L14-05	1.39–1.69	COL3746.1.1	Poales, *Carex*, Amblystegiaceae	10,079	50	11,611.5	222
L14-05	5.00–5.17	COL3747.1.1	fine organic bulk material	1259	34	1226.5	55
L14-05	5.83–6.05	COL3748.1.1	Poales	54,619	2314	n. c.	n. c.
L14-05	7.75–7.89	COL3623.1.1	mosses (*Drepanocladus revolvens*)	51,136	1743	n. c.	n. c.
L14-02	0.31–0.44	COL3614.1.1	*Carex* stem	>modern	n.c.	n. c.	n.c.
L14-02	1.70–1.86	COL3616.1.1	fine root remains	33,082	281	37,307.5	893.5
L14-02	2.06–2.23	COL3733.1.1	Poales, roots, lignified remains	33,711	247	37,977.5	754
L14-02	3.47–3.77	COL3617.1.1	wood	43,354	727	46,719.5	1508.5
L14-02	4.03–4.16	COL3618.1.1	wood	44,245	807	47,619.5	1753.5
L14-02	4.27–4.48	COL3619.1.1	fine root remains	43,802	811	47,194.5	1727.5
L14-02	4.62–4.78	COL3620.1.1	fine root remains	42,939	715	46,310.0	1429.0
L14-02	5.33–5.56	COL3621.1.1	fine root remains	62,773	7099	n. c.	n. c.
L14-02	6.17–6.32	COL3734.1.1	roots	51,479	1594	n. c.	n. c.
L14-02	7.28–7.48	COL3732.1.1	fine organic bulk material, rootlets, Poales	51,509	1597	n. c.	n. c.
L14-02	7.48–7.68	COL3735.1.1	*Carex*, lignified remains, rootlets	54,594	2354	n. c.	n. c.
L14-02	8.12–8.29	COL3730.1.1	*Carex*, lignified remains, rootlets	61,056	5081	n. c.	n. c.
L14-02	8.51–8.68	COL3731.1.1	*Carex*, lignified remains, rootlets	56,379	2866	n. c.	n. c.
L14-02	9.02–9.28	COL3736.1.1	lignified remains, roots, remains of higher plants	54,430	2275	n. c.	n. c.
L14-02	9.84–10.12	COL3737.1.1	roots, fine organic bulk material	53,591	2049	n. c.	n. c.
L14-02	10.39–10.64	COL3738.1.1	roots, fine organic bulk material	51,995	1702	n. c.	n. c.
L14-02	10.92–11.04	COL3739.1.1	Poales	49,309	1235	n. c.	n. c.
L14-03	0.24–0.50	COL3740.1.1	lignified remains, fine organic bulk material	3975	36	4454.5	73
L14-03	0.8–0.95	COL3741.1.1	fine organic bulk material, roots	29,259	174	33,433	411
L14-03	1.86–1.98	COL3742.1.1	lignified remains, fine organic bulk material, Amblystegiaceae	52,072	1704	n. c.	n. c.
L14-03	2.30–2.42	COL3743.1.1	wood	58,311	3628	n. c.	n. c.

**Table 2:** *Larix* primer pairs with the corresponding annealing temperature (Ta), product size, the possible single-nucleotide polymorphism (SNP) variants and ecoPCR specificity indicated by possible matches (MF: mismatches in forward primer; MR: mismatches in reverse primer; and L: length of DNA target sequence).

Primer Name	Sequence (5’–3’)	Ta (°C)	Product Size (nt)	SNP Variants	ecoPCR Specificity
cp77444F cp77444R	GGAAGGAAGGCGGAATGAATAG TCCATTACAGTACTTCCCTTCAG	58	37	A/C	*Larix decidua*
					(MF = 0, MR = 0, L = 37)
					*Larix occidentalis*
					(MF = 0, MR = 0, L = 38)
					*Pseudotsuga sinensis*
					(MF = 2, MR = 1, L = 37)
cp103595F cp103595R	GATGGATCACTTTCTGTCAAGGT GTATAGACAATGTTTCTCTGGCG	58	54	T/C	*Larix decidua*
					(MF = 0, MR = 0, L = 54)
					*Pseudotsuga menziesii*
					(MF = 0, MR = 2, L = 54)
					*Pseudotsuga sinensis*
					(MF = 0, MR = 2, L = 54)
					*Picea morrisonicola*
					(MF = 0, MR = 3, L = 54)
					*Picea abies*
					(MF = 0, MR = 3, L = 54)
					*Picea jezoensis*
					(MF = 0, MR = 3, L = 54)
					*Keteleeria*
					(MF = 0, MR = 3, L = 54)
					*Cedrus deodara*
					(MF = 0, MR = 3, L = 54)
					*Keteleeria davidiana*
					(MF = 0, MR = 3, L =54)
					*Abies koreana*
					(MF = 0, MR = 3, L = 54)
					*Abies nephrolepis*
					(MF = 0, MR = 3, L = 54)
					*Picea glauca*
					(MF = 0, MR = 3, L = 54)
					*Tsuga chinensis*
					(MF = 0, MR = 3, L = 54)
					*Pseudolarix amabilis*
					(MF = 0, MR = 3, L = 54)

**Table 3:** List of samples containing *Larix* sequences in the metabarcoding data, with their extraction negative controls (EN), sorted by the core or hand-pieces (HP) from which they were collected. PCR and cloning success (+: successful, −: not successful) are given, as is the number of retrieved sequences for each sample and the detected SNP variants of the two markers cp77444 and cp103595 including their abundance. If PCRs were not successful, the samples were not cloned (n.c.).

Core/HP	Sample Depth	PCR-Product cp77444	PCR-Product cp103595	Cloning Success cp77444	Cloning Success cp103595	No. of Retrieved Sequences cp77444	Detected Variants cp77444	No. of Retrieved Sequences cp103595	Detected Variants cp103595
L14-02	7.53 m	+	+	+	-	0	-	-	-
-	EN2	-	-	n. c.	n.c.	-	-	-	-
L14-03	6.72 m	+	+	+	+	0	-	13	T
-	EN3	-	-	n.c.	n.c.	-	-	-	-
L14-03	7.59 m	+	-	+	n.c.	11	A	-	-
L14-03	8.56 m	+	+	+	+	8	A	7	3× C, 4× T
L14-03	9.61 m	-	-	n.c.	n.c.	-	-	-	-
L14-03	10.11 m	-	-	n.c.	n.c.	-	-	-	-
L14-03	11.77 m	+	+	+	+	9	A	3	T
-	EN5	-	-	n.c.	n.c.	-	-	-	-
L14-05	3.3 m	-	+	+	+	1	A	1	T
L14-05	3.7 m	-	-	n.c.	n.c.	-	-	-	-
-	EN6	-	-	n.c.	n.c.	-	-	-	-
L14-04	6.15 m	-	+	n.c.	+	-	-	2	C
L14-04	7.27 m	+	+	+	+	7	A	13	T
-	EN7	-	-	n.c.	n.c.	-	-	-	-
L14-04	8.03 m	+	+	+	+	7	3× C, 4× A	7	1× C, 6× T
L14-04C	2.05 m	+	+	+	+	9	A	11	C
L14-04C	3.15 m	+	+	+	+	10	A	15	8× C, 7× T
L14-04C	3.55 m	+	+	+	+	6	A	13	2× C, 11× T
-	EN8	-	-	n.c.	n.c.	-	-	-	-
-	NTCs	-	-	n.c.	n.c.	-	-	-	-

## Supplementary Materials

The following are available online at PANGAEA (https://doi.pangaea.de/10.1594/PANGAEA.878888): Supplement 1.docx (1. extraction negative controls, 2. PCA biplots); Supplement 2.xlsx: Table S1: Terrestrial vascular plant sedaDNA sequence counts, Table S2: Extraction negative control EH312, Table S3: Extraction negative control EH322, Table S4: Extraction negative control EH342, Table S5: Terrestrial vascular plant pollen counts.

## References

[1] Shackleton N. (1967). Oxygen isotope analyses and pleistocene temperatures re-assessed. Nature.

[2] Mix A.C., Ruddiman W.F. (1984). Oxygen-isotope analyses and Pleistocene ice volumes. Quat. Res..

[3] Lambeck K., Esat T.M., Potter E.-K. (2002). Links between climate and sea levels for the past three million years. Nature.

[4] Andreev A.A., Grosse G., Schirrmeister L., Kuznetsova T.V., Kuzmina S.A., Bobrov A.A., Tarasov P.E., Novenko E.Y., Meyer H., Derevyagin A.Y. (2009). Weichselian and Holocene palaeoenvironmental history of the Bol’shoy Lyakhovsky Island, New Siberian Archipelago, Arctic Siberia. Boreas.

[5] Andreev A.A., Schirrmeister L., Tarasov P.E., Ganopolski A., Brovkin V., Siegert C., Wetterich S., Hubberten H.-W. (2011). Vegetation and climate history in the Laptev Sea region (Arctic Siberia) during Late Quaternary inferred from pollen records. Quat. Sci. Rev..

[6] Kienast F., Wetterich S., Kuzmina S., Schirrmeister L., Andreev A.A., Tarasov P., Nazarova L., Kossler A., Frolova L., Kunitsky V.V. (2011). Paleontological records indicate the occurrence of open woodlands in a dry inland climate at the present-day Arctic coast in western Beringia during the Last Interglacial. Quat. Sci. Rev..

[7] Wetterich S., Schirrmeister L., Andreev A.A., Pudenz M., Plessen B., Meyer H., Kunitsky V.V. (2009). Eemian and Late Glacial/Holocene palaeoenvironmental records from permafrost sequences at the Dmitry Laptev Strait (NE Siberia, Russia). Palaeogeogr. Palaeoclimatol. Palaeoecol..

[8] Wetterich S., Tumskoy V., Rudaya N., Andreev A.A., Opel T., Meyer H., Schirrmeister L., Hüls M. (2014). Ice Complex formation in arctic East Siberia during the MIS3 Interstadial. Quat. Sci. Rev..

[9] Emiliani C. (1955). Pleistocene Temperatures. J. Geol..

[10] Lisiecki L.E., Raymo M.E. (2005). A Pliocene-Pleistocene stack of 57 globally distributed benthic δ ^18^ O records: Pliocene-Pleistocene benthic stack. Paleoceanography.

[11] Railsback L.B., Gibbard P.L., Head M.J., Voarintsoa N.R.G., Toucanne S. (2015). An optimized scheme of lettered marine isotope substages for the last 1.0 million years, and the climatostratigraphic nature of isotope stages and substages. Quat. Sci. Rev..

[12] Hewitt G.M. (1996). Some genetic consequences of ice ages, and their role in divergence and speciation. Biol. J. Linn. Soc..

[13] Hewitt G.M. (2000). The genetic legacy of the Quaternary ice ages. Nature.

[14] Payette S., Lavoie C. (1994). The arctic tree line as a record of past and recent climatic changes. Environ. Rev..

[15] Arctic Climate Impact Assessment (2004). Arctic Climate Impact Assessment Impacts of a Warming Arctic.

[16] Andersen K.K., Azuma N., Barnola J.-M., Bigler M., Biscaye P., Caillon N., Chappellaz J., Clausen H.B., Dahl-Jensen D., Fischer H. (2004). High-resolution record of Northern Hemisphere climate extending into the last interglacial period. Nature.

[17] Woodward F.I., Lomas M.R., Betts R.A. (1998). Vegetation-climate feedbacks in a greenhouse world. Philos. Trans. R. Soc. Lond. B Biol. Sci..

[18] MacDonald G., Kremenetski K., Beilman D. (2008). Climate change and the northern Russian treeline zone. Philos. Trans. R. Soc. B Biol. Sci..

[19] Levis S., Foley J.A., Pollard D. (2000). Large-Scale Vegetation Feedbacks on a Doubled CO_2_ Climate. J. Clim..

[20] Levis S., Foley J.A., Pollard D. (1999). Potential high-latitude vegetation feedbacks on CO_2_-induced climate change. Geophys. Res. Lett..

[21] Foley J.A., Costa M.H., Delire C., Ramankutty N., Snyder P. (2003). Green surprise? How terrestrial ecosystems could affect earth’s climate. Front. Ecol. Environ..

[22] Bonan G.B. (2008). Forests and Climate Change: Forcings, Feedbacks, and the Climate Benefits of Forests. Science.

[23] Kruse S., Wieczorek M., Jeltsch F., Herzschuh U. (2016). Treeline dynamics in Siberia under changing climates as inferred from an individual-based model for *Larix*. Ecol. Model..

[24] Herzschuh U., Birks H.J.B., Laepple T., Andreev A., Melles M., Brigham-Grette J. (2016). Glacial legacies on interglacial vegetation at the Pliocene-Pleistocene transition in NE Asia. Nat. Commun..

[25] Andreev A.A., Grosse G., Schirrmeister L., Kuzmina S.A., Novenko E.Y., Bobrov A.A., Tarasov P.E., Ilyashuk B.P., Kuznetsova T.V., Krbetschek M. (2004). Late Saalian and Eemian palaeoenvironmental history of the Bol’shoy Lyakhovsky Island (Laptev Sea region, Arctic Siberia). Boreas.

[26] Kienast F., Tarasov P., Schirrmeister L., Grosse G., Andreev A.A. (2008). Continental climate in the East Siberian Arctic during the last interglacial: Implications from palaeobotanical records. Glob. Planet. Chang..

[27] Willerslev E., Hansen A.J., Binladen J., Brand T.B., Gilbert M.T.P., Shapiro B., Bunce M., Wiuf C., Gilichinsky D.A., Cooper A. (2003). Diverse Plant and Animal Genetic Records from Holocene and Pleistocene Sediments. Science.

[28] Willerslev E., Davison J., Moora M., Zobel M., Coissac E., Edwards M.E., Lorenzen E.D., Vestergard M., Gussarova G., Haile J. (2014). Fifty thousand years of Arctic vegetation and megafaunal diet. Nature.

[29] Jørgensen T., Haile J., Möller P., Andreev A., Boessenkool S., Rasmussen M., Kienast F., Coissac E., Taberlet P., Brochmann C. (2012). A comparative study of ancient sedimentary DNA, pollen and macrofossils from permafrost sediments of northern Siberia reveals long-term vegetational stability. Mol. Ecol..

[30] Zimmermann H.H., Raschke E., Epp L.S., Stoof-Leichsenring K.R., Schwamborn G., Schirrmeister L., Overduin P.P., Herzschuh U. (2017). Sedimentary ancient DNA and pollen reveal the composition of plant organic matter in Late Quaternary permafrost sediments of the Buor Khaya Peninsula (north-eastern Siberia). Biogeosciences.

[31] Willerslev E., Cappellini E., Boomsma W., Nielsen R., Hebsgaard M.B., Brand T.B., Hofreiter M., Bunce M., Poinar H.N., Dahl-Jensen D. (2007). Ancient biomolecules from deep ice cores reveal a forested Southern Greenland. Science.

[32] Parducci L., Matetovici I., Fontana S.L., Bennett K.D., Suyama Y., Haile J., Kjaer K.H., Larsen N.K., Drouzas A.D., Willerslev E. (2013). Molecular- and pollen-based vegetation analysis in lake sediments from central Scandinavia. Mol. Ecol..

[33] Parducci L., Väliranta M., Salonen J.S., Ronkainen T., Matetovici I., Fontana S.L., Eskola T., Sarala P., Suyama Y. (2015). Proxy comparison in ancient peat sediments: Pollen, macrofossil and plant DNA. Philos. Trans. R. Soc. Lond. B. Biol. Sci..

[34] Pedersen M.W., Ginolhac A., Orlando L., Olsen J., Andersen K., Holm J., Funder S., Willerslev E., Kjær K.H. (2013). A comparative study of ancient environmental DNA to pollen and macrofossils from lake sediments reveals taxonomic overlap and additional plant taxa. Quat. Sci. Rev..

[35] Van der Knaap W.O. (1987). Transported pollen and spores on Spitsbergen and Jan Mayen. Pollen Spores.

[36] Birks H.H., Smol J.P., Birks H.J.B., Last W.M., Bradley R.S., Alverson K. (2001). Plant macrofossils. Tracking Environmental Change Using Lake Sediments.

[37] Niemeyer B., Klemm J., Pestryakova L.A., Herzschuh U. (2015). Relative pollen productivity estimates for common taxa of the northern Siberian Arctic. Rev. Palaeobot. Palynol..

[38] Campbell I.D., McDonald K., Flannigan M.D., Kringayark J. (1999). Long-distance transport of pollen into the Arctic. Nature.

[39] Li Y., Xu Q., Yang X., Chen H., Lu X. (2005). Pollen-vegetation relationship and pollen preservation on the Northeastern Qinghai-Tibetan Plateau. Grana.

[40] De Klerk P., Donner N., Joosten H., Karpov N.S., Minke M., Seifert N., Theuerkauf M. (2009). Vegetation patterns, recent pollen deposition and distribution of non-pollen palynomorphs in a polygon mire near Chokurdakh (NE Yakutia, NE Siberia). Boreas.

[41] Sønstebø J.H., Gielly L., Brysting A.K., Elven R., Edwards M., Haile J., Willerslev E., Coissac E., Rioux D., Sannier J. (2010). Using next-generation sequencing for molecular reconstruction of past Arctic vegetation and climate. Mol. Ecol. Resour..

[42] Niemeyer B., Epp L.S., Stoof-Leichsenring K.R., Pestryakova L.A., Herzschuh U. (2017). A comparison of sedimentary DNA and pollen from lake sediments in recording vegetation composition at the Siberian treeline. Mol. Ecol. Resour..

[43] Parducci L., Suyama Y., Lascoux M., Bennett K.D. (2005). Ancient DNA from pollen: A genetic record of population history in Scots pine. Mol. Ecol..

[44] Coissac E., Riaz T., Puillandre N. (2012). Bioinformatic challenges for DNA metabarcoding of plants and animals. Mol. Ecol..

[45] Taberlet P., Coissac E., Pompanon F., Gielly L., Miquel C., Valentini A., Vermat T., Corthier G., Brochmann C., Willerslev E. (2007). Power and limitations of the chloroplast trnL (UAA) intron for plant DNA barcoding. Nucleic Acids Res..

[46] Grigoriev M.N. (2008). Cryomorphogenesis and Lithodynamics of the Nearshore Shelf Zone of the East Siberian Seas.

[47] Hubberten H.W., Andreev A., Astakhov V.I., Demidov I., Dowdeswell J.A., Henriksen M., Hjort C., Houmark-Nielsen M., Jakobsson M., Kuzmina S. (2004). The periglacial climate and environment in northern Eurasia during the last glaciation. Quat. Sci. Rev..

[48] Schwamborn G., Wetterich S. Russian-German cooperation CARBOPERM: Field campaigns to Bol’shoy Lyakhovsky Island in 2004. http://epic.awi.de/37311/.

[49] Rivas-Martínez S., Rivas-Sáenz S. (1996). Worldwide Bioclimatic Classification System.

[50] CAVM Team (2003). Circumpolar Arctic Vegetation Map (1:7,500,000 scale), Conservation of Arctic Flora and Fauna (CAFF) Map No. 1..

[51] Opel T., Wetterich S., Meyer H., Dereviagin A.Y., Fuchs M.C., Schirrmeister L. (2017). Ground-ice stable isotopes and cryostratigraphy reflect late Quaternary palaeoclimate in the Northeast Siberian Arctic (Oyogos Yar coast, Dmitry Laptev Strait). Clim. Past.

[52] Ilyashuk B.P., Andreev A.A., Bobrov A.A., Tumskoy V.E., Ilyashuk E.A. (2006). Interglacial History of a Palaeo-lake and Regional Environment: A Multi-proxy Study of a Permafrost Deposit from Bol’shoy Lyakhovsky Island, Arctic Siberia. J. Paleolimnol..

[53] Rethemeyer J., Fülöp R.-H., Höfle S., Wacker L., Heinze S., Hajdas I., Patt U., König S., Stapper B., Dewald A. (2013). Status report on sample preparation facilities for 14C analysis at the new CologneAMS center. Nucl. Instrum. Methods Phys. Res. Sect. B Beam Interact. Mater. At..

[54] Schirrmeister L., Schwamborn G., Overduin P.P., Strauss J., Fuchs M.C., Grigoriev M., Yakshina I., Rethemeyer J., Dietze E., Wetterich S. (2017). Yedoma Ice Complex of the Buor Khaya Peninsula (southern Laptev Sea). Biogeosciences.

[55] Stuiver M., Polach H.A. (1977). Discussion Reporting of 14C Data. Radiocarbon.

[56] Reimer P.J., Bard E., Bayliss A., Beck J.W., Blackwell P.G., Ramsey C.B., Buck C.E., Cheng H., Edwards R.L., Friedrich M. (2013). IntCal13 and Marine13 radiocarbon age calibration curves 0–50,000 years cal BP. Radiocarbon.

[57] Stuiver M., Reimer P.J., Reimer R.W. CALIB 7.1. http://calib.org.

[58] Zimmermann H.H., Harms L., Trense D., Epp L.S., Stoof-Leichsenring K.R., Frickenhaus S., Pestryakova L., Herzschuh U. Genetic variation of larches at the Siberian tundra-taiga ecotone inferred from the assembly of chloroplast genomes and mitochondrial sequences. BMC Genom..

[59] Untergasser A., Cutcutache I., Koressaar T., Ye J., Faircloth B.C., Remm M., Rozen S.G. (2012). Primer3—New capabilities and interfaces. Nucleic Acids Res..

[60] Ficetola G.F., Coissac E., Zundel S., Riaz T., Shehzad W., Bessière J., Taberlet P., Pompanon F. (2010). An In silico approach for the evaluation of DNA barcodes. BMC Genom..

[61] Boyer F., Mercier C., Bonin A., Le Bras Y., Taberlet P., Coissac E. (2016). OBITools: A Unix-inspired software package for DNA metabarcoding. Mol. Ecol. Resour..

[62] Katoh K., Misawa K., Kuma K., Miyata T. (2002). MAFFT: A novel method for rapid multiple sequence alignment based on fast Fourier transform. Nucleic Acids Res..

[63] Kearse M., Moir R., Wilson A., Stones-Havas S., Cheung M., Sturrock S., Buxton S., Cooper A., Markowitz S., Duran C. (2012). Geneious Basic: An integrated and extendable desktop software platform for the organization and analysis of sequence data. Bioinformatics.

[64] Altschul S.F., Gish W., Miller W., Myers E.W., Lipman D.J. (1990). Basic local alignment search tool. J. Mol. Biol..

[65] Andrews S. (2010). FastQC: A Quality Control Tool for High Throughput Sequence Data.

[66] Epp L.S., Gussarova G., Boessenkool S., Olsen J., Haile J., Schrøder-Nielsen A., Ludikova A., Hassel K., Stenøien H.K., Funder S. (2015). Lake sediment multi-taxon DNA from North Greenland records early post-glacial appearance of vascular plants and accurately tracks environmental changes. Quat. Sci. Rev..

[67] Soininen E.M., Gauthier G., Bilodeau F., Berteaux D., Gielly L., Taberlet P., GUssarova G., Bellemain E., Hassel K., Stenøien H.K. (2015). Highly overlapping winter diet in two sympatric lemming species revealed by DNA metabarcoding. PLoS ONE.

[68] Kanz C., Aldebert P., Althorpe N., Baker W., Baldwin A., Bates K., Browne P., van den Broek A., Castro M., Cochrane G. (2005). The EMBL nucleotide sequence database. Nucleic Acids Res..

[69] Sayers E.W., Barrett T., Benson D.A., Bryant S.H., Canese K., Chetvernin V., Church D.M., DiCuccio M., Edgar R., Federhen S. (2009). Database resources of the National Center for Biotechnology Information. Nucleic Acids Res..

[70] Epp L.S., Boessenkool S., Bellemain E.P., Haile J., Esposito A., Riaz T., Erséus C., Gusarov V.I., Edwards M.E., Johnsen A. (2012). New environmental metabarcodes for analysing soil DNA: Potential for studying past and present ecosystems. Mol. Ecol..

[71] Schnell I.B., Bohmann K., Gilbert M.T.P. (2015). Tag jumps illuminated—Reducing sequence-to-sample misidentifications in metabarcoding studies. Mol. Ecol. Resour..

[72] Faegri K., Iversen J., Faegri K., Kaland P.E., Krzywinski K. (1989). Textbook of Pollen Analysis.

[73] Stockmarr J. (1971). Tablets with spores used in absolute pollen analysis. Pollen Spores.

[74] Beug H.-J. (2004). Leitfaden der Pollenbestimmung.

[75] Kupriyanova L.A., Alyoshina L.A. (1972). Pollen and Spores of Plants in the Flora of the European Part of the USSR (Vol. 1).

[76] Kupriyanova L.A., Alyoshina L.A. (1978). Pollen and Spores of Plants from the Flora of European Part of USSR. Lamiaceae-Zygophyllaceae.

[77] Moore P.D., Webb J.A., Collison M.E. (1991). Pollen Analysis.

[78] Savelieva L.A., Raschke E.A., Titova D.V. (2013). Photographic Atlas of Plants and Pollen of the Lena River Delta.

[79] Sokolovskaya A.P. (1958). Vegetation of Far North and its development. Pollen of the Arctic Plants.

[80] Van Geel B., Smol J.P., Birks H.J.B., Last W.M., Bradley R.S., Alverson K. (2001). Non-pollen palynomorphs. Volume 3: Terrestrial, algal and silicaceous indicators. Tracking Environmental Change Using Lake Sediments: Terrestrial, Algal, and Siliceous Indicators.

[81] Van Geel B., Hallewas D.P., Pals J.P. (1983). A late Holocene deposit under the Westfriese Zeedijk near Enkhuizen (Prov. of Noord-Holland, The Netherlands): Palaeoecological and archaeological aspects. Rev. Palaeobot. Palynol..

[82] Van Geel B., Aptroot A. (2006). Fossil ascomycetes in Quaternary deposits. Nova Hedwig..

[83] Jankovská V., Komárek J. (2000). Indicative value of Pediastrum and other coccal green algae in palaeoecology. Folia Geobot..

[84] Komárek J., Jankovská V. (2001). Review of the Green Algal Genus Pediastrum; Implication for Pollenanalytical Research.

[85] (2003). The Angiosperm Phylogeny Group An update of the Angiosperm Phylogeny Group classification for the orders and families of flowering plants: APG II. Bot. J. Linn. Soc..

[86] Hurlbert S.H. (1971). The nonconcept of species diversity: A critique and alternative parameters. Ecology.

[87] Heck K.L., van Belle G., Simberloff D. (1975). Explicit calculation of the rarefaction diversity measurement and the determination of sufficient sample size. Ecology.

[88] R Core Team (2014). R: A Language and Environment for Statistical Computing.

[89] Oksanen J., Blanchet F.G., Kindt R., Legendre P., Minchin P.R., O’Hara R.B., Simpson G.L., Solymos P., Stevens M.H.H., Wagner H. (2011). Vegan: Community Ecology Package. R Package Version 2.0–2..

[90] Juggins S. (2012). Rioja: Analysis of Quaternary Science Data, R Package Version 0.7–3.

[91] Simpson G.L. (2007). Analogue methods in palaeoecology: Using the analogue package. J. Stat. Softw..

[92] Simpson G.L., Oksanen J. (2016). Analogue: Analogue and Weighted Averaging Methods for Palaeoecology.

[93] Zimmermann H.H., Raschke E., Epp L.S., Stoof-Leichsenring K.R., Schirrmeister L., Schwamborn G., Herzschuh U. Ancient DNA Dataset, Pollen and Non-Pollen Palynomorphs of Four Permafrost Cores Hand-Pieces, Bol’shoy Lyakhovsky Island. https://doi.pangaea.de/10.1594/PANGAEA.878888.

[94] Wetterich S., Tumskoy V., Rudaya N., Kuznetsov V., Maksimov F., Opel T., Meyer H., Andreev A.A., Schirrmeister L. (2016). Ice Complex permafrost of MIS5 age in the Dmitry Laptev Strait coastal region (East Siberian Arctic). Quat. Sci. Rev..

[95] French H.M. (2007). The Periglacial Environment.

[96] Sjögren P., van der Knaap W.O., Huusko A., van Leeuwen J.F.N. (2008). Pollen productivity, dispersal, and correction factors for major tree taxa in the Swiss Alps based on pollen-trap results. Rev. Palaeobot. Palynol..

[97] Edwards M.E., Brubaker L.B., Lozhkin A.V., Anderson P.M. (2005). Structurally Novel Biomes: A Response to Past Warming in Beringia. Ecology.

[98] Van Geel B., Protopopov A., Protopopova V., Pavlov I., van der Plicht J., van Reenen G.B.A. (2016). Larix during the Mid-Pleniglacial (Greenland Interstadial 8) on Kotelny Island, northern Siberia. Boreas.

[99] Brubaker L.B., Anderson P.M., Edwards M.E., Lozhkin A.V. (2005). Beringia as a glacial refugium for boreal trees and shrubs: New perspectives from mapped pollen data. J. Biogeogr..

[100] Campbell I.D. (1999). Quaternary pollen taphonomy: Examples of differential redeposition and differential preservation. Palaeogeogr. Palaeoclimatol. Palaeoecol..

[101] Eisenhut G. (1961). Untersuchungen über die Morphologie und Ökologie der Pollenkörner Heimischer und Fremdländischer Waldbäume.

[102] Parducci L., Jørgensen T., Tollefsrud M.M., Elverland E., Alm T., Fontana S.L., Bennett K.D., Haile J., Matetovici I., Suyama Y. (2012). Glacial survival of boreal trees in northern Scandinavia. Science.

[103] Parducci L., Edwards M.E., Bennett K.D., Alm T., Elverland E., Tollefsrud M.M., Jørgensen T., Houmark-Nielsen M., Larsen N.K., Kjær K.H. (2012). Response to Comment on “Glacial Survival of Boreal Trees in Northern Scandinavia”. Science.

[104] Koltowski Z., Pluta S., Jablonski B., Szklanowska K. (1999). Pollination requirements of eight cultivars of black currant (Ribes nigrum L.). J. Hortic. Sci. Biotechnol..

[105] Faegri K., van der Pijl L. (1980). The Principles of Pollination Ecology, Third revised edition.

[106] Iversen C.M., Sloan V.L., Sullivan P.F., Euskirchen E.S., McGuire A.D., Norby R.J., Walker A.P., Warren J.M., Wullschleger S.D. (2015). The unseen iceberg: Plant roots in arctic tundra. New Phytol..

[107] Polezhaeva M.A., Lascoux M., Semerikov V.L. (2010). Cytoplasmic DNA variation and biogeography of *Larix* Mill. in Northeast Asia. Mol. Ecol..

[108] Semerikov V.L., Semerikova S.A., Polezhaeva M.A., Kosintsev P.A., Lascoux M. (2013). Southern montane populations did not contribute to the recolonization of West Siberian Plain by Siberian larch (*Larix sibirica*): A range-wide analysis of cytoplasmic markers. Mol. Ecol..

[109] Kharuk V.I., Ranson K.J., Im S.T., Naurzbaev M.M. (2006). Forest-tundra larch forests and climatic trends. Russ. J. Ecol..

[110] Köppen W., Köppen W., Geiger R. (1936). Das geografische System der Klimate. Handbuch der Klimatologie.

[111] Crawford R.M.M., Jeffree C.E., Rees W.G. (2003). Paludification and forest retreat in northern oceanic environments. Ann. Bot..

[112] Iwasaki H., Saito H., Kuwao K., Maximov T.C., Hasegawa S. (2010). Forest decline caused by high soil water conditions in a permafrost region. Hydrol. Earth Syst. Sci..

[113] Abaimov A.P. (2010). Geographical distribution and genetics of Siberian larch species. Permafrost Ecosystems: Siberian Larch Forests.

